# Brain‐wide associations between white matter and age highlight the role of fornix microstructure in brain ageing

**DOI:** 10.1002/hbm.26333

**Published:** 2023-05-17

**Authors:** Max Korbmacher, Ann Marie de Lange, Dennis van der Meer, Dani Beck, Eli Eikefjord, Arvid Lundervold, Ole A. Andreassen, Lars T. Westlye, Ivan I. Maximov

**Affiliations:** ^1^ Department of Health and Functioning Western Norway University of Applied Sciences Bergen Norway; ^2^ NORMENT Centre for Psychosis Research, Division of Mental Health and Addiction University of Oslo and Oslo University Hospital Oslo Norway; ^3^ Mohn Medical Imaging and Visualisation Center (MMIV) Bergen Norway; ^4^ Department of Psychiatry University of Oxford Oxford UK; ^5^ LREN, Centre for Research in Neurosciences–Department of Clinical Neurosciences CHUV and University of Lausanne Lausanne Switzerland; ^6^ Faculty of Health, Medicine and Life Sciences Maastricht University Maastricht Netherlands; ^7^ Department of Psychiatric Research, Diakonhjemmet Hospital Oslo Norway; ^8^ Department of Psychology University of Oslo Oslo Norway; ^9^ Department of Radiology Haukeland University Hospital Bergen Norway; ^10^ Department of Biomedicine University of Bergen Bergen Norway; ^11^ KG Jebsen Centre for Neurodevelopmental Disorders University of Oslo Oslo Norway

**Keywords:** ageing, brain age, diffusion, fornix, magnetic resonance imaging, white matter, forceps

## Abstract

Unveiling the details of white matter (WM) maturation throughout ageing is a fundamental question for understanding the ageing brain. In an extensive comparison of brain age predictions and age‐associations of WM features from different diffusion approaches, we analyzed UK Biobank diffusion magnetic resonance imaging (dMRI) data across midlife and older age (*N* = 35,749, 44.6–82.8 years of age). Conventional and advanced dMRI approaches were consistent in predicting brain age. WM‐age associations indicate a steady microstructure degeneration with increasing age from midlife to older ages. Brain age was estimated best when combining diffusion approaches, showing different aspects of WM contributing to brain age. Fornix was found as the central region for brain age predictions across diffusion approaches in complement to forceps minor as another important region. These regions exhibited a general pattern of positive associations with age for intra axonal water fractions, axial, radial diffusivities, and negative relationships with age for mean diffusivities, fractional anisotropy, kurtosis. We encourage the application of multiple dMRI approaches for detailed insights into WM, and the further investigation of fornix and forceps as potential biomarkers of brain age and ageing.

## Graphical abstract

- Conventional and advanced diffusion MRI approaches predict brain age consistently, yet best when combined.
- Fornix and forceps minor were central regions for these predictions across diffusion approaches.
- We present general patterns of white matter deterioration for higher ages in fornix, forceps minor, and across the brain.

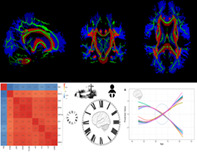

## 1 INTRODUCTION

Along the past decades, neuroscientific research, and particularly magnetic resonance imaging (MRI) have increased our understanding of the biological mechanisms associated with brain tissue maturation and ageing effects (Grady, 2012; Symms et al., 2004; Wrigglesworth et al., 2021). A fundamental basis for that are large‐scale MRI databases, such as UK Biobank (UKB; Sudlow et al., 2015) or the Human Connectome Project (Van Essen et al., 2012), allowing one to provide larger generalizability for revealed effects (Marek et al., 2022). Simultaneously, large‐scale data provide sufficient power for the application of advanced multivariate statistical models, and machine learning (ML) techniques. Brain age prediction is an example of such technique, translating large amounts of complex multidimensional data into practically interpretable outputs. Brain age prediction involves training a ML model to determine trajectories of brain ageing from a series of brain MRI features. Once the model is trained, it can predict the age of brains not included in the training data. The disparity between chronological age and predicted age, the so‐called brain age gap (BAG), can be used as an indicator of various disorders and potentially general health status (Beck et al., 2022; Cole et al., 2017; Franke & Ten Gaser, 2019; Kaufmann et al., 2019; Leonardsen et al., 2021). For example, BAG has been associated with stroke history, diabetes, smoking, alcohol intake, several cognitive measures (Cole, 2020; Leonardsen et al., 2021), cardiovascular risk factors (Beck et al., 2022), stroke risk (de Lange et al., 2020), and loneliness (de Lange et al., 2021), mortality risk, different brain and psychiatric disorders, particularly Alzheimer's disease and schizophrenia (Cole & Franke, 2017; Franke & Ten Gaser, 2019; Kaufmann et al., 2019; Rokicki et al., 2021). Yet, the effect of brain age on brain maturation remains unclear (Vidal‐Pineiro et al., 2021), indicating the need for further investigation.

BAG and age trajectories offer paths toward a better understanding of the ageing brain. There are various detectable age‐related brain changes, such as GM and white matter (WM) atrophy (Lawrence et al., 2021), WM de‐differentiation (Cox et al., 2016a), and functional connectivity changes (Wrigglesworth et al., 2021) which have hence informed the choice of brain‐age modeling‐parameters (Beck et al., 2021; Beck et al., 2022; Cole, 2020; de Lange et al., 2020; Le Chen et al., 2020; Richard et al., 2018; Salih et al., 2021). In that context, many ML approaches have been used to make robust and clinically relevant brain age predictions from different MRI modalities (Baecker et al., 2021; Dosenbach et al., 2010; Franke et al., 2010; Kaufmann et al., 2019); yet, particularly the eXtreme Gradient Boosting (Chen & Guestrin, 2016) regressor model, using a decision tree approach, is increasingly used for brain age predictions from large‐scale data due to its precision and speed (Beck et al., 2021; de Lange et al., 2019; Kaufmann et al., 2019). Especially diffusion magnetic resonance imaging (dMRI) and structural MRI have been shown useful for brain age predictions (Beck et al., 2021; Beck et al., 2022; Cole, 2020; de Lange et al., 2020; Le Chen et al., 2020; Richard et al., 2018; Salih et al., 2021). However, further systematic, sufficiently powered assessments of dMRI‐derived brain age and how diffusion metrics map onto age are needed. To this end, there are only a few publications about the influence of diffusion derived metrics on brain age predictions. Moreover, studies on the relationships between age and diffusion metrics usually focus on diffusion tensor imaging (DTI; Basser et al., 1994). In turn, advanced dMRI approaches (Fieremans et al., 2011; Jensen et al., 2005; Kaden et al., 2016a; Kaden et al., 2016b; Novikov et al., 2019; Reisert et al., 2017; Westlye et al., 2010) which offer additional details on WM microstructure and, hence, brain maturation processes require further research. In order to address this shortcoming, this study focusses in dMRI‐derived measures from a large midlife‐to‐older adult sample and the measures' associations with age.

DMRI‐derived measures consist of unique parameters allowing both to reveal WM changes at micrometer scale and to provide the basis for a prediction of macroscopic outcomes, such as age. Conventionally, WM brain architecture is described using DTI (Basser et al., 1994). However, recent advances offer more biophysically meaningful approaches (Novikov et al., 2019), and sensible foundation for cross‐validation and better comparability (Beck et al., 2021). DTI‐derived measures, namely fractional anisotropy (FA), and axial (AD), mean (MD), and radial (RD) diffusivity have all been shown to be highly age sensitive (Beck et al., 2021; Cox et al., 2016a; Westlye et al., 2010). Nevertheless, the DTI approach is limited by the Gaussian diffusion assumption and is unable to take into account entangled WM microstructure features (Beck et al., 2021). In the present work, we consider (1) the Bayesian rotationally invariant approach (BRIA; Reisert et al., 2017), (2) diffusion kurtosis imaging (DKI; Jensen et al., 2005), (3) kurtosis derived supplement, known as *white matter tract integrity* (WMTI; Fieremans et al., 2011) (4) spherical mean technique (SMT; Kaden et al., 2016a), and (5) *multi‐compartment spherical mean technique* (mcSMT; Kaden et al., 2016b) in addition to DTI. Only a few studies have compared dMRI models directly as original brain age predictors (Beck et al., 2021; Maximov et al., 2021; Raghavan et al., 2021). Yet, brain age and age curve assessments of DTI, BRIA, DKI, WMTI, SMT, mcSMT (Table S10) in a representative sample present a great interest, as well as most influential WM regions for brain ageing. Our assessments focus on the process of ageing (from midlife to late adulthood), starting by associating BAG across diffusion approaches and compare–predicted versus chronological‐age correlations in order to assess predictors' consistency. As fornix was identified as most contributing feature in these predictions, and forceps minor as another influential region, post‐hoc analyses focused on both fornix, forceps minor, and whole‐brain relationships with age. Fornix was the strongest correlate of age, and fornix and forceps minor features were highly correlated across approaches. Finally, we created fornix, forceps minor, and whole‐brain‐age curves expecting curvilinear relationships reflecting brain‐tissue‐composition at different ageing stages (Beck et al., 2021; Davis et al., 2009; Westlye et al., 2010).

## 2 METHODS

### 2.1 Sample characteristics

**FIGURE 1:**
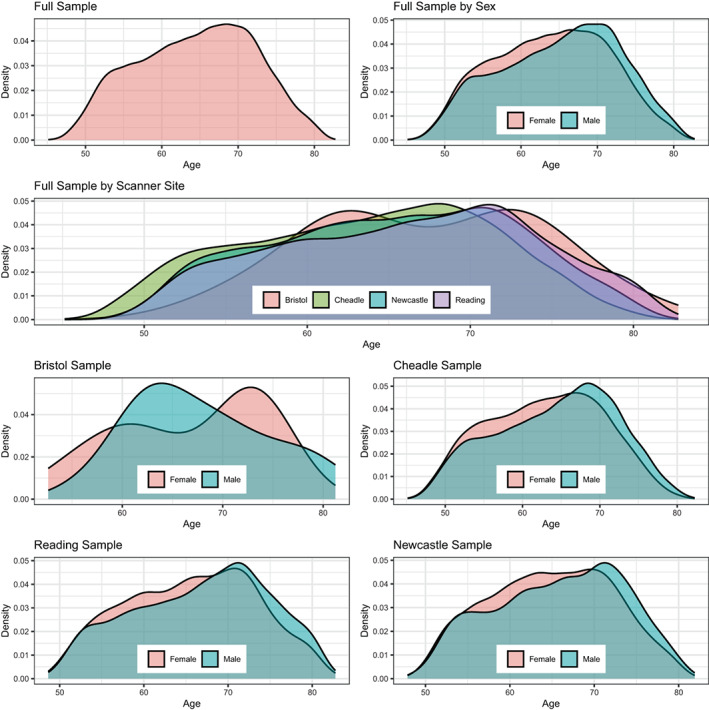
Density plots for the sample's age by sex and scanner site. The *y*‐axis indicates the probability of age scaled to 1.

The original UKB (Sudlow et al., 2015) diffusion MRI data consisted of *N* = 42,208 participants. After exclusions, based on later withdrawn consent and an ICD‐10 diagnosis from categories F, G, I, and stroke (excluded: *N* = 3521), and data sets not meeting quality control standards (*N* = 2938) using the YTTRIUM method (Maximov et al., 2021), we obtained a final sample consisting of 35,749 healthy adults (age range 44.57–82.75, Mage = 64.46, SDage = 7.62, Mdage = 64.97; 52.96% females, 47.04% males). In brief, YTTRIUM converts diffusion scalar metric into 2D format using a structural similarity extension (Wang et al., 2004) of each scalar map to their mean image in order to create a 2D distribution of image and diffusion parameters. The quality check is based on a two‐step clustering algorithm applied to identify subjects located out of the main distribution. We define healthy here as the absence of mental and behavioral disorder (ICD‐10 category F), disease of the nervous system (ICD‐10 category G), and disease of the circulatory system (ICD‐10 category I). Included participants showed generally higher cognitive test performance and took less medication than excluded subjects (Table 1). Participants were recruited and scanned at four different sites: 57.62% in Cheadle, 26.30% in Newcastle, 15.96% in Reading, and 0.12% in Bristol (Figure 1). Imbalances in age distributions in the Bristol sample can be attributed to the small number of participants sampled (*N* = 43).

**TABLE 1:** Included and excluded sample characteristics.

Variable	Excluded (*N* = 6459)	Included (*N* = 35,749)	*P*‐value	Cohens *d*
Number of medications	2.812 (2.782)	1.784 (2.034)	<.001	0.474
Self‐rated health	2.204 (0.764)	1.965 (0.644)	<.001	0.360
Number of correctly solved matrix puzzles	7.671 (2.191)	8.012 (2.126)	<.001	−0.159
Number of correctly solved tower puzzles	9.650 (3.318)	9.917 (3.224)	<.001	−0.083
Number of correct symbol digit matches	17.808 (5.414)	18.998 (5.246)	<.001	−0.226
Number of incorrectly matched pairs	2.239 (1.282)	2.215 (1.274)	0.250	0.019
Matrix puzzle response time in seconds	81.116 (16.605)	83.011 (15.873)	<.001	−0.119
Maximum number of remembered digits	6.497 (1.642)	6.678 (1.538)	<.001	−0.117
Fluid intelligence	6.429 (2.096)	6.634 (2.054)	<.001	−0.099
Prospective memory score	1.069 (0.433)	1.068 (0.397)	0.783	0.004

*Note*: Mean (SD) for each sample's variables. *p*‐values are indicated for Welch two sample t‐tests.

### 2.2 MRI acquisition, diffusion pipeline, and tract‐based spatial statistic analysis

UKB MRI data acquisition procedures are described elsewhere (Miller et al., 2016; Sudlow et al., 2015). The brain scan protocol (https://biobank.ctsu.ox.ac.uk/crystal/refer.cgi?id=2367) was applied at each scanner site (see also documentation: https://biobank.ctsu.ox.ac.uk/crystal/refer.cgi?id=1977). Shortly, the diffusion protocol consists of two b‐values (1000 and 2000 s/mm^2^) with 50 noncoplanar diffusion weighting gradients per each shell. For a susceptibility artefact correction, nondiffusion weighted images with an opposite gradient encoding direction were acquitted as well.

Diffusion data preprocessing was conducted as described in Maximov et al. (2019), using an optimized pipeline which includes corrections for noise (Veraart et al., 2016), Gibbs ringing (Kellner et al., 2016), susceptibility‐induced and motion distortions, and eddy current artefacts (Andersson & Sotiropoulos, 2016). Isotropic Gaussian smoothing was carried out with the FSL (Jenkinson et al., 2012) function *fslmaths* with a Gaussian kernel of 1 mm^3^. After that DTI, DKI, and WMTI metrics were estimated using Matlab 2017b (Mathworks, 2017). Employing the multishell data, DKI and WMTI metrics were estimated using Matlab code (https://github.com/NYU-DiffusionMRI/DESIGNER; Fieremans et al., 2011). SMT, and mcSMT metrics were estimated using original code (https://github.com/ekaden/smt; Kaden et al., 2016a), as well as Bayesian estimates/BRIA were estimated by the original Matlab code (https://bitbucket.org/reisert/baydiff/src/master/; Reisert et al., 2017).

In total, we obtained 28 metrics from 6 diffusion approaches (DTI, DKI, WMTI, SMT, mcSMT, BRIA; Beck et al., 2021; Kaden et al., 2016b; Maximov et al., 2019; Benitez et al., 2018; Hope et al., 2019; Pines et al., 2020). In order to normalize all metrics, we used TBSS (Smith et al., 2006), as part of FSL (Smith et al., 2004). In brief, initially all BET‐extracted (Smith, 2002) FA images were aligned to MNI space using nonlinear transformation (FNIRT; Jenkinson et al., 2012). Afterward, the mean FA image and related mean FA skeleton were derived. Each diffusion scalar map was projected onto the mean FA skeleton using the TBSS procedure. In order to provide a quantitative description of diffusion metrics we evaluated averaged values over the skeleton and two white matter atlases, namely the JHU atlas (Mori & Wakana, 2005) and the JHU tractographic atlas (Hua et al., 2008). Finally, we obtained 20 WM tracts and 48 regions of interest (ROIs) based on a probabilistic white matter atlas (JHU; Hua et al., 2008) for each of the 28 metrics, including the mean skeleton values. Altogether, 1932 features per individual were derived (28 metrics * [48 ROIs +1 skeleton mean + 20 tracts]; see number of dMRI features in Table 2)). We included both whole‐brain average metrics in addition to tracts and regional averages, as these provide spatially differential information (Figure S16), also expressed the metrics' relationships with age (Barrick et al., 2010; Beck et al., 2021; Eikenes et al., 2023; Kochunov et al., 2007; Westlye et al., 2010).

**TABLE 2:** Performance of brain age prediction models.

Approach^a^	Number of MRI features	*R* ^2^ (SD)	RMSE (SD)	MAE (SD)	Prediction‐age correlation* [95% CI]	Corrected prediction‐age correlation* [95% CI]
BRIA	690	0.550 (0.012)	5.007 (0.057)	4.002 (0.042)	0.742 [0.737, 0.747]	0.892^b^ [0.889, 0.894]
DKI	207	0.576 (0.015)	4.958 (0.077)	3.975 (0.068)	0.754 [0.755, 0.764]	0.903 [0.901, 0.905]
DTI	276	0.571 (0.014)	4.983 (0.072)	3.984 (0.062)	0.756 [0.751, 0.761]	0.900 [0.897, 0.902]
SMT	276	0.531 (0.010)	5.214 (0.053)	4.183 (0.036)	0.729 [0.724, 0.734]	0.899 [0.897, 0.901]
mcSMT	276	0.519 (0.011)	5.175 (0.045)	4.153 (0.036)	0.721 [0.716, 0.726]	0.892^b^ [0.889, 0.894]
WMTI	207	0.585 (0.012)	4.903 (0.065)	3.928 (0.050)	0.765 [0.761, 0.770]	0.902 [0.900, 0.904]
Mean multimodal	28	0.393 (0.012)	5.932 (0.051)	4.812 (0.046)	0.627 [0.621, 0.634]	0.905 [0.903, 0.907]
Full multimodal	1932	0.645 (0.011)	4.534 (0.041)	3.624 (0.037)	0.804 [0.800, 0.808]	0.907 [0.905, 0.909]

*Note*: *R* ^2^, RMSE, MAE are displayed in the format Mean (Standard Deviation), Pearson's correlations are displayed in the format Correlation Score 95% Confidence Interval (Lower Bound, Upper Bound). Mean multimodal refers to diffusion metrics averaged over the skeleton for all six diffusion approaches. Full multimodal refers to all diffusion data from the six diffusion approaches, that is, mean multimodal data in addition to metrics averaged over the JHU atlas regions.

Abbreviations: BRIA, Bayesian rotationally invariant approach; MAE = mean absolute error; *R* ^2^ = variance explained; RMSE = root mean squared error.

^a^ For an overview of the metrics contained in each of the diffusion approaches see Table S10.

^b^ Details on the smallest correlation: BRIA Corrected Prediction‐Age Correlation *r* = 0.89173, mcSMT Corrected Prediction‐Age Correlation *r* = 0.89176.

* All correlation were significant at *p* < .001.

### 2.3 Brain age predictions

**FIGURE 2:**
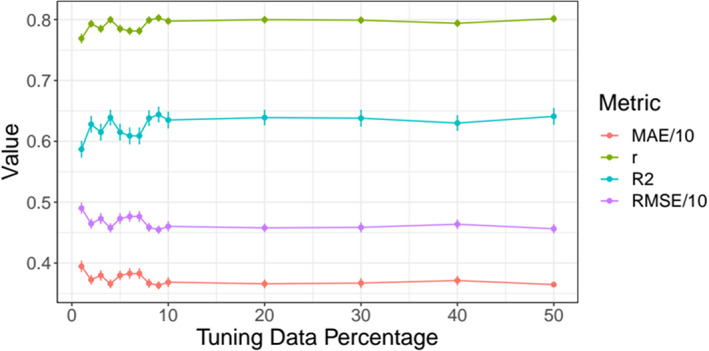
Model performance for different train‐test splits. Model metrics *R* ^2^, root mean squared error (RMSE), mean absolute error (MAE) and their standard deviations, as well as the Pearson's correlations between predicted and chronological age and its 95% confidence interval are displayed for different training data percentages of the total data (x‐axis). For visualization purposes, RMSE and MAE were divided by 10. For exact values see Table S1.

First, brain age predictions were performed using XGBoost (Chen & Guestrin, 2016) in Python (v3.7.1). To evaluate how much data was needed for hyper‐parameter tuning while accurately predicting brain age from all 1932 brain features, we divided the full dataset (*N* = 35,749) into two equal parts: one validation set and one hyper‐parameter tuning set for independent parameter‐tuning. From the hyper‐parameter tuning set, data was randomly sampled into subsamples consisting of 358, 715, 1073, 1430, 1788, 2145, 2503, 2860, 3218, 3575, 7150, 10,725, 14,300, or 17,875 participants, corresponding to 1%, 2%, 3%, 4%, 5%, 6%, 7%, 8%, 9%, 10%, 20%, 30%, 40%, and 50% of the total subjects, respectively (Figure 2). Hyper‐parameters were tuned on these sub‐samples and then tested on the remaining half, that is, the validation sample, using 10‐fold cross validation showing model performance to not further improve past the 10% (tuning) data mark, informing our tuning‐validation‐split (Figure 2, Table S1, trained models in S2).

Second, in order to compare the different diffusion approaches, based on the previous steps, the training‐test split was fixed at previously used 10% training data (*N* = 3575) and 90% test data (*N* = 32,174) which indicated a best fit at a learning rate = 0.05, max layers/depth = 3, and number of trees = 750. These tuned parameters were used for 10‐fold cross‐validations brain age predictions on the test data of all six individual models, one multimodal model combining all metrics from all diffusion models, and one multimodal model using only mean values from all diffusion models (Table 2).

Third, uncorrected BAG was calculated as the difference between chronological age Ω and predicted age *P*:

(1) $${\mathrm{BAG}}_{u}=P-\Omega$$

We calculated BAG as it is the commonly used metric indicative of general health when using brain age predictions (Beck et al., 2022; Cole, 2020; Cole et al., 2017; Cole & Franke, 2017; de Lange et al., 2020; de Lange et al., 2021; Franke & Ten Gaser, 2019; Kaufmann et al., 2019; Leonardsen et al., 2021; Rokicki et al., 2021; Vidal‐Pineiro et al., 2021). BAG is, however, sensitive to the age distribution of the sample (de Lange et al., 2019; de Lange & Cole, 2020). Hence as a supplement, age‐bias‐corrected predicted age was calculated from the intercept and slope of age predictions as previously described (de Lange et al., 2019; de Lange & Cole, 2020):

(2) P=α×Ω+β

(3) $$\text{BAGc}=\left(P+\left[\Omega -\left(\alpha \times \Omega +\beta \right)\right]\right)-\Omega$$

**FIGURE 3:**
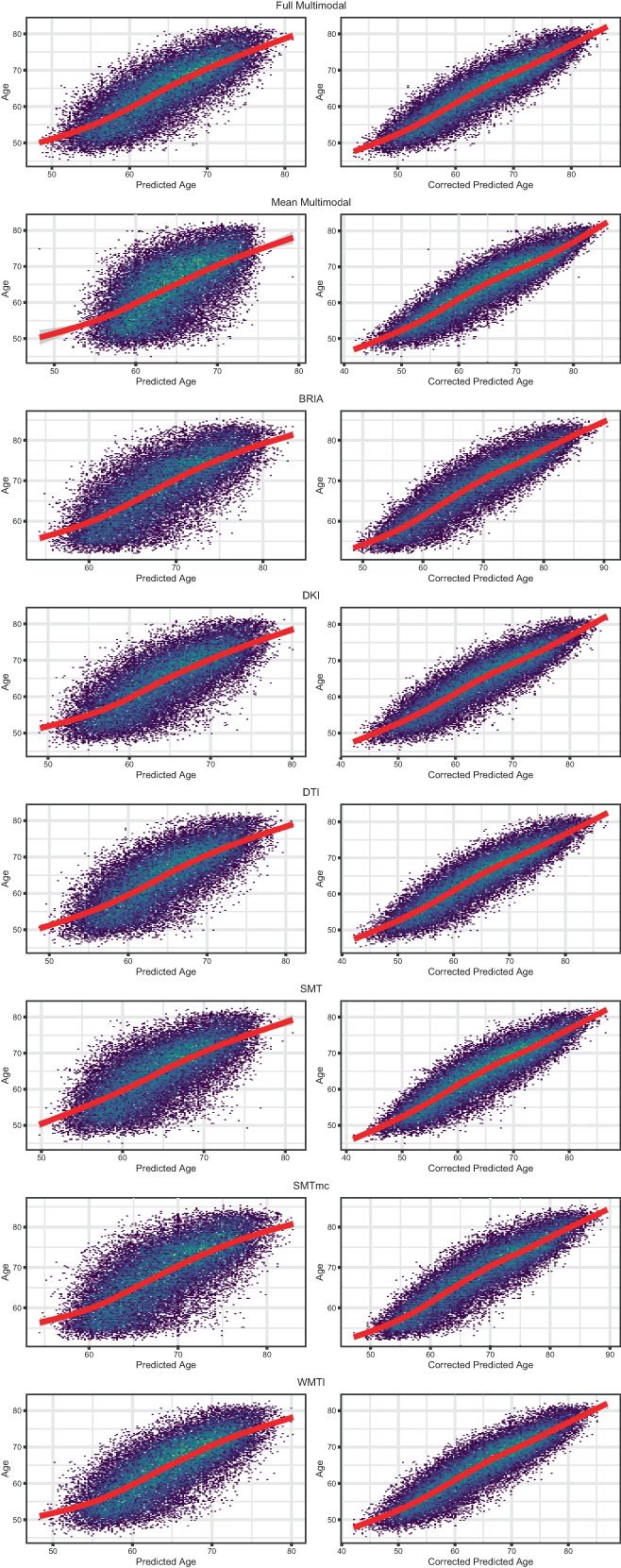
Corrected and uncorrected brain age by age for each of the utilized brain age models.

P represents predicted age modelled from chronological age *Ω*, with intercept *β* and slope *α*. This age‐bias correction allowed for a bias‐corrected BAG estimate (BAGc). See Figure 3 for both uncorrected and age‐bias‐corrected brain ages over age.

### 2.4 Statistical analyses

All statistical analyses were carried out using R (v3.6.0; www.r-project.org/). *p*‐values were adjusted for multiple comparison using Holm correction (Holm, 1979). Model performance for brain ages estimations across different diffusion approaches are presented in addition to top five features for each brain age model ranked based on their model contributions (variance explained, as determined by permutation feature importance testing). Then, the correlation structure of age, brain age, BAG, and brain features (identified as main contributors in the model and whole‐brain‐average scores) were examined across diffusion approaches. In detail: first, brain ages were correlated across diffusion‐approach‐specific brain ages. Then, the correlations between true and estimated age across diffusion approaches were compared. Second, BAGs were correlated across diffusion approaches. Third, we present the correlation structure of fornix and age, and present brain‐age crude and adjusted age‐relationships for all included metrics (*M*).

(4) $$M=\beta 0+\beta \text{1Age}+\beta {\text{2Age}}^{2}+\beta 3\times {\text{Site}}^{*}\mathrm{Sex}+\beta {\text{4Sex}}^{*}\mathrm{Age}+\beta \text{5Sex}+\beta \text{6Site}$$

Fourth, we plot absolute/crude whole‐brain and fornix diffusion metrics by age, and contrast these with diffusion metrics (*M*) adjusted for age, sex, and site. To test the age‐sensitivity of the metrics, we removed age from the model and compared the models using Likelihood Ratio tests.

(5) $$M=\beta 0+\beta {\text{1Site}}^{*}\mathrm{Sex}+\beta \text{2Sex}+\beta \text{3Site}$$

**FIGURE 4:**
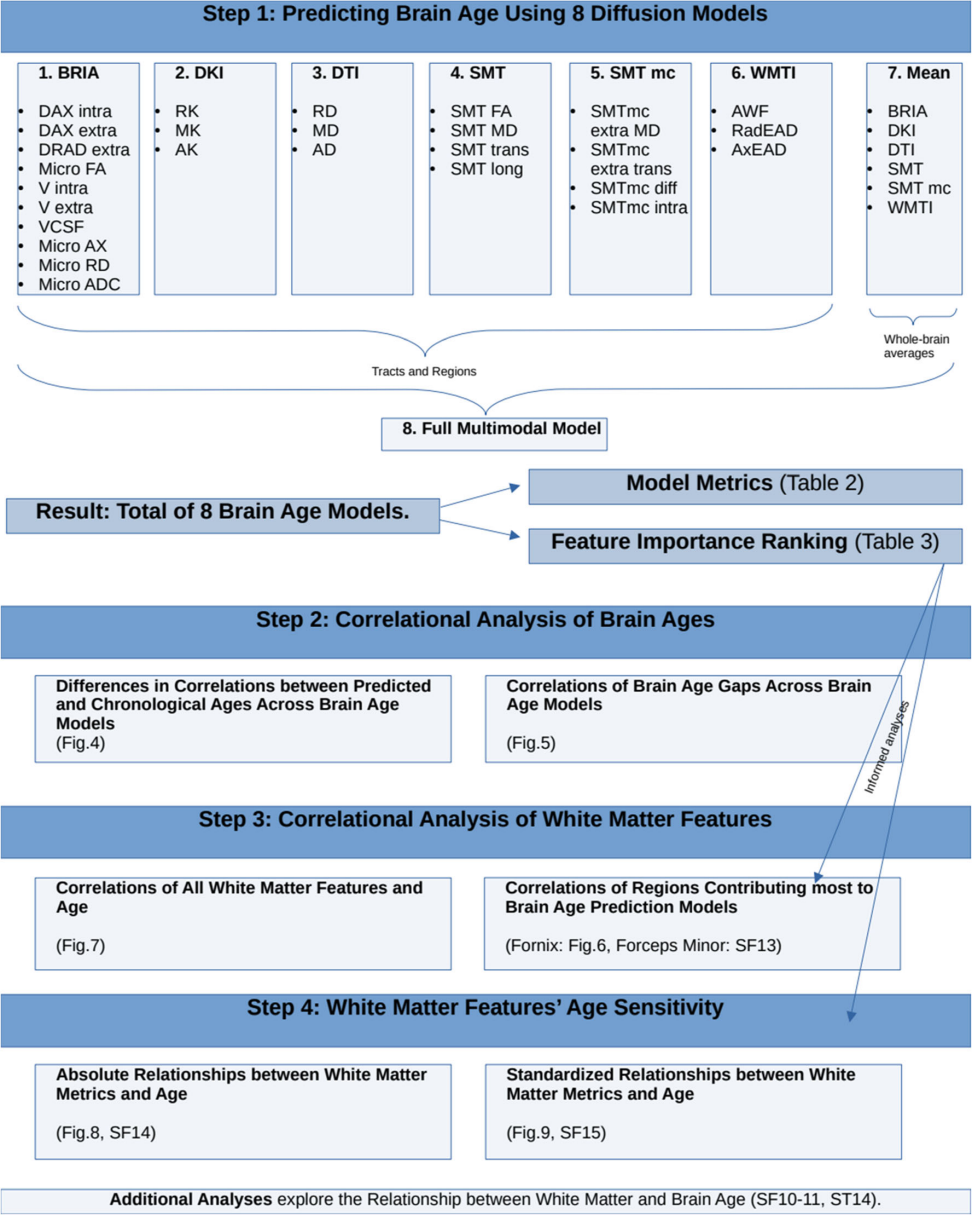
Overview of the analysis steps.

We also assess to which extent the regression lines can be called linear by comparing model fit of generalized additive models with simple linear regression models for fornix and whole brain features. Finally, we associate the first two principal components of all WM features with the different brain ages to assess the relationship between BAG and WM. For an overview of the analyses see Figure 4.

## 3 RESULTS

### 3.1 Brain age predictions

Table 2 presents a comparison between different diffusion approaches in predicting brain age for each diffusion approach. The strongest correlation between uncorrected age predictions and chronological age was observed for WMTI Pearson's *r* = 0.765, 95% CI [0.761, 0.770], *p* < .001, and the smallest for mcSMT Pearson's *r* = 0.721, 95% CI [0.716, 0.726], *p* < .001.

**FIGURE 5:**
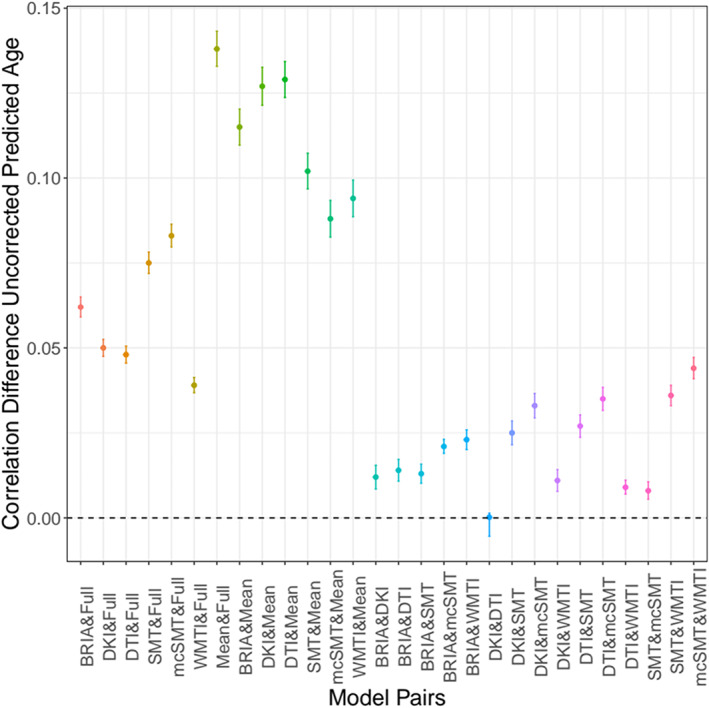
Differences between Pearson's correlations of chronological and uncorrected predicted ages across diffusion approaches with 95% confidence interval. Differences between Pearson's correlation coefficients of chronological and uncorrected predicted age by diffusion approach. See Figure S8 for correlational differences between approaches for corrected brain age predictions.

Hotelling's (Hotelling, 1936) *t*‐tests were used to compare correlations between uncorrected predicted age and chronological age across diffusion models. Zou's (Zou, 2007) method was used to estimate the confidence intervals around the correlation differences (Figure 5 and Table S3; Figure S8 and Table S2 for corrected prediction correlation comparisons). These differences were not significantly different from each other for model pairs DKI and DTI (*p ≈* 1). All other correlations were different from each other, Pearson's *rs*diff ≤0.15, *p* < .001, with the biggest difference observed between mean and full multimodal scores’ correlations (Table S2 for exact values).

Permutation feature importance estimates across diffusion models showed that fornix contributed strongest to variance explained (Table 3), which was in correspondence with feature rankings by gain score (XBGoost Developers, 2021; Table S15). Follow‐up models which had fornix features removed had lower model fit, explained less variance in age, and predicted‐chronological‐age correlations were smaller than for models containing fornix (*rs*_*diff*_ < −0.003, *ps* < .001; Table S16). Another potentially important region was the forceps minor, also contributing significantly to age predictions (Table 3).

**TABLE 3:** Top five diffusion metrics ranked by their contribution to variance explained (*R* ^2^) in age.

BRIA	DKI	DTI	SMT	mcSMT	WMTI	Multimodal
Micro FA fornix 0.1954 ± 0.0027	AK right anterior limb of internal capsule 0.0984 ± 0.0014	MD fornix 0.0712 ± 0.0013	MD fornix 0.0795 ± 0.0018	Extratrans fornix 0.0498 ± 0.0013	AWF fornix 0.1699 ± 0.0023	Micro FA fornix 0.0914 ± 0.0011
Vextra forceps minor 0.0278 ± 0.0007	RK fornix 0.0884 ± 0.0016	FA forceps minor 0.0533 ± 0.0011	FA right superior longitudinal fasciculus 0.0267 ± 0.0007	Intra forceps minor 0.0444 ± 0.0009	radEAD fornix to right striaterminalis 0.0283 ± 0.0007	AK anterior limb of internal capsule 0.0055 ± 0.0011
Vextra body of the corpus callosum 0.0261 ± 0.0007	MK left external capsule 0.0259 ± 0.0006	RD fornix to right Striaterminalis 0.0462 ± 0.0009	Longitudinal fornix 0.0251 ± 0.0006	Intra fornix 0.0289 ± 0.0009	AWF forceps minor 0.0194 ± 0.0005	FA forceps minor 0.0219 ± 0.0006
Micro FA fornix to right Striaterminalis 0.0203 ± 0.0006	MK right superior longitudinal fasciculus 0.0214 ± 0.0006	FA right superior cerebellar peduncle 0.0221 ± 0.0006	Trans fornix to right striaterminalis 0.0204 ± 0.0006	Extratrans fornix to right Striaterminalis 0.0201 ± 0.0006	axEAD forceps minor 0.0193 ± 0.0007	RD right fornix stria terminalis 0.0214 ± 0.0006
Vintra right superior cerebellar peduncle 0.0194 ± 0.0006	RK forceps minor 0.0208 ± 0.0005	FA body of the corpus callosum 0.0218 ± 0.0006	FA fornix 0.0192 ± 0.0006	Extratrans right external capsule 0.0163 ± 0.0007	axEAD left posterior limb of internal capsule 0.0173 ± 0.0006	AK Genu corpus callosum 0.0095 ± 0.0003

*Note*: Variance explained (*R* ^2^) by a single feature refers here to the part of the total variance explained by the respective feature in each of the brain age models presented in Table 2. Multimodal refers to an approach using the diffusion metrics from all diffusion approaches. Cells containing fornix are marked in green. Cells containing forceps minor are marked in blue. See Table S19 for an overview of all the features and their variance explained.

Abbreviations: BRIA, Bayesian rotationally invariant approach; DKI, diffusion kurtosis imaging; DTI, diffusion tensor imaging; mcSMT, multicompartment spherical mean technique; SMT, spherical mean technique; WMTI, white matter tract integrity.

### 3.2 BAG across diffusion approaches and age

**FIGURE 6:**
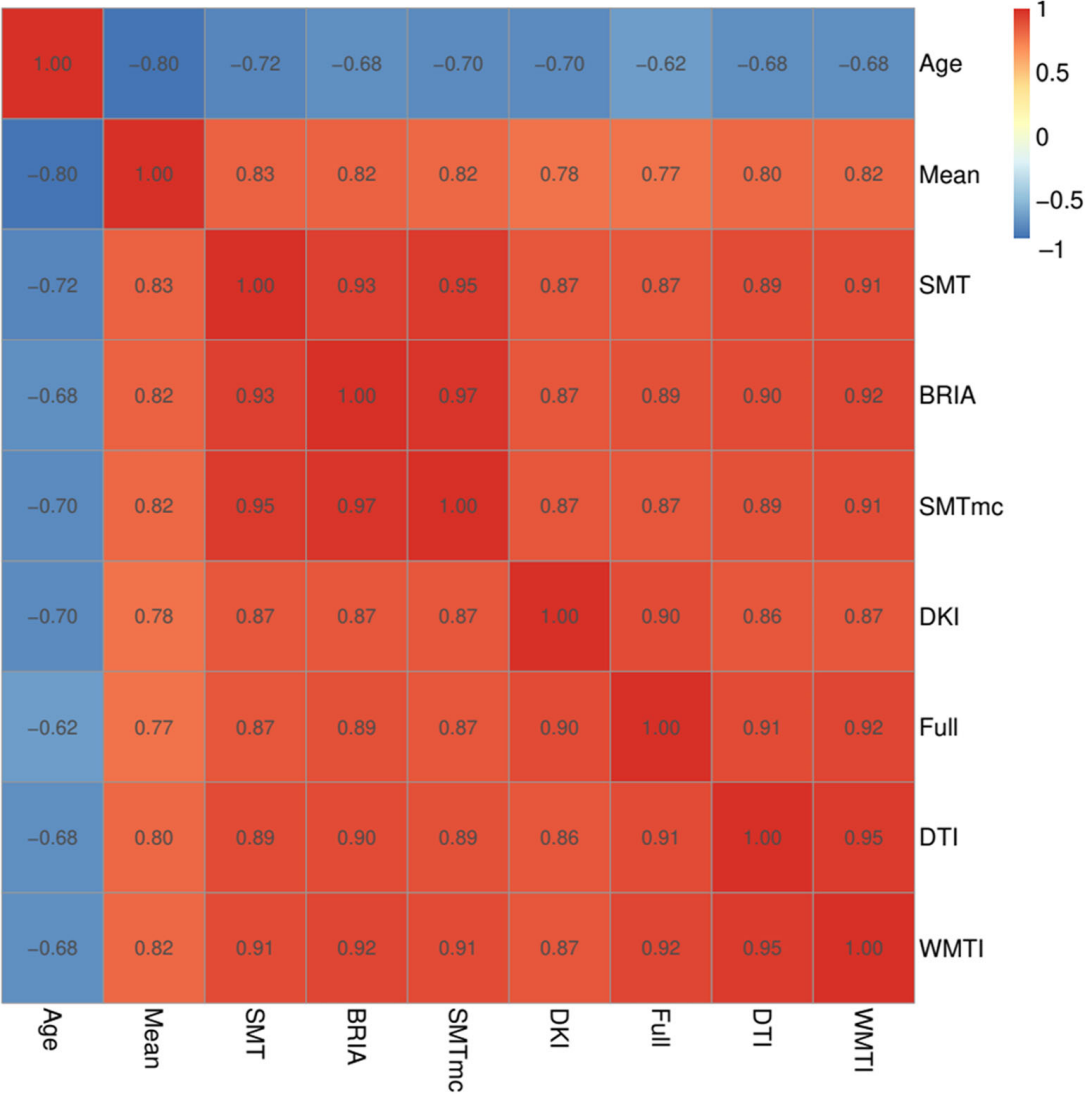
Correlations of uncorrected BAG and age across used diffusion approaches. Age‐BAG correlations were significant at *pHolm* < .001. For the corrected BAG correlations across models see Figure S1.

In order to compare uncorrected BAG (BAGu) calculations across the used diffusion approaches, BAGu was correlated from different diffusion approaches and with age. Correlations between the six diffusion approaches ranged between *r* = 0.857 and *r* = 0.966 (Figures 6 and S1 for corrected BAG correlations). Overall, BAGu scores from the different approaches were strongest related to WMTI BAGc (range: *r* = 0.873–0.952), and weakest to mean multimodal BAGu (range: *r* = 0.779–0.828), and could be observed in one cluster containing DKI, DTI, WMTI, and multimodal BAGu and a second cluster containing BRIA, SMT, and SMTmc. However, DKI, BAGu was more strongly correlated with full multimodal BAGc than with other well‐performing approaches DTI (Pearson's *r*diff = 0.03, *p* < .001) and WMTI (*r*diff = 0.03, *p* < .001). Vice versa, DTI BAGc correlated strongest with WMTI BAGc (*r* = 0.905, *p* < .001).

### 3.3 Associations between diffusion metrics and age

**FIGURE 7:**
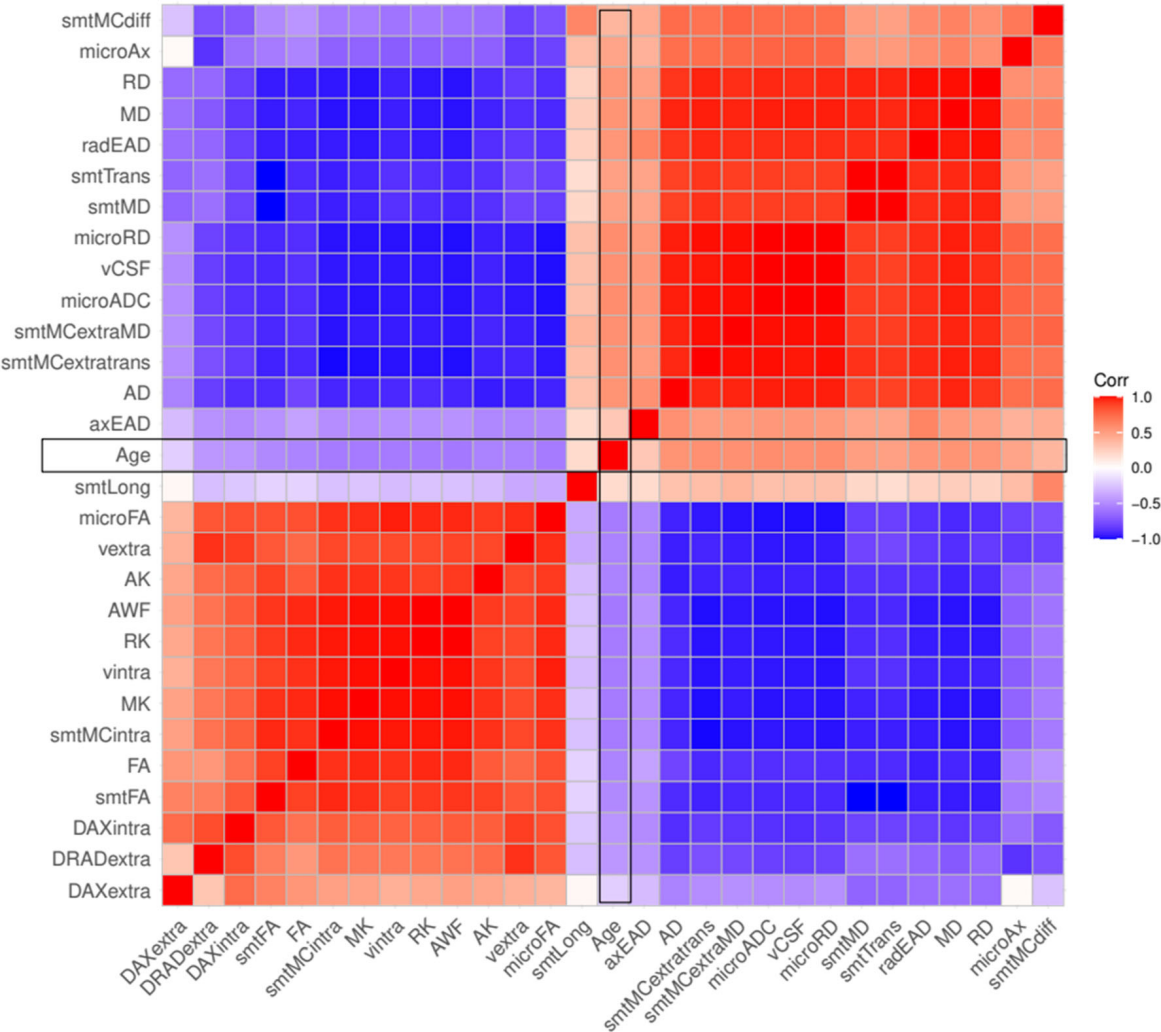
Correlation matrix for fornix diffusion metrics and chronological age. All correlations were significant at Holm‐corrected *pHolm* < .05.

A correlational analysis was used to demonstrate associations among fornix diffusion metrics and age (Figure 7, including QC outliers: Figure S4). Association strengths ranged from to *r* = −0.997 (smtTrans and smtMCintra) to *r* = 0.999 (smtTrans and smtMD). Correlations between fornix metrics and age ranged from *r* = −0.558 (smtMCintra) to *r* = 0.570 (microRD), and between forceps minor metrics and age from *r* = −0.519 (FA) to *r* = 0.493 (RD, see Figure S13).

**FIGURE 8:**
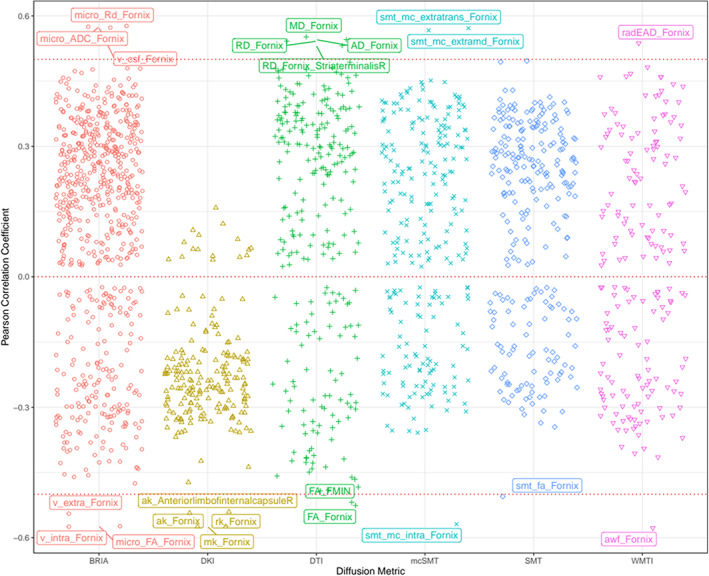
Correlations between diffusion metrics and age. Each point indicates one correlation between a diffusion metric and chronological age. Names of diffusion metrics are displayed when correlations between the metric and age reached a Pearson correlation of |*r|* > 0.5. Holm correction (Holm, 1979) was used for Holm‐correction, and all displayed values were significant at *p* < .001. For the distribution of the correlations see Figure S12.

Correlations across all diffusion metrics and age (1933 × 1933 correlations), age‐fornix associations were the strongest (Figure 8, Figure S12). Overall, the significant *N* = 1823 correlations (at *p*_*Holm*_ < .001) ranged from |*r*| = 0.024 to |*r*| = 0.578 with |*r*|_Mean_ = 0.245, |*r*|_SD_ = 0.122.

### 3.4 Age trajectories of diffusion features

**FIGURE 9:**
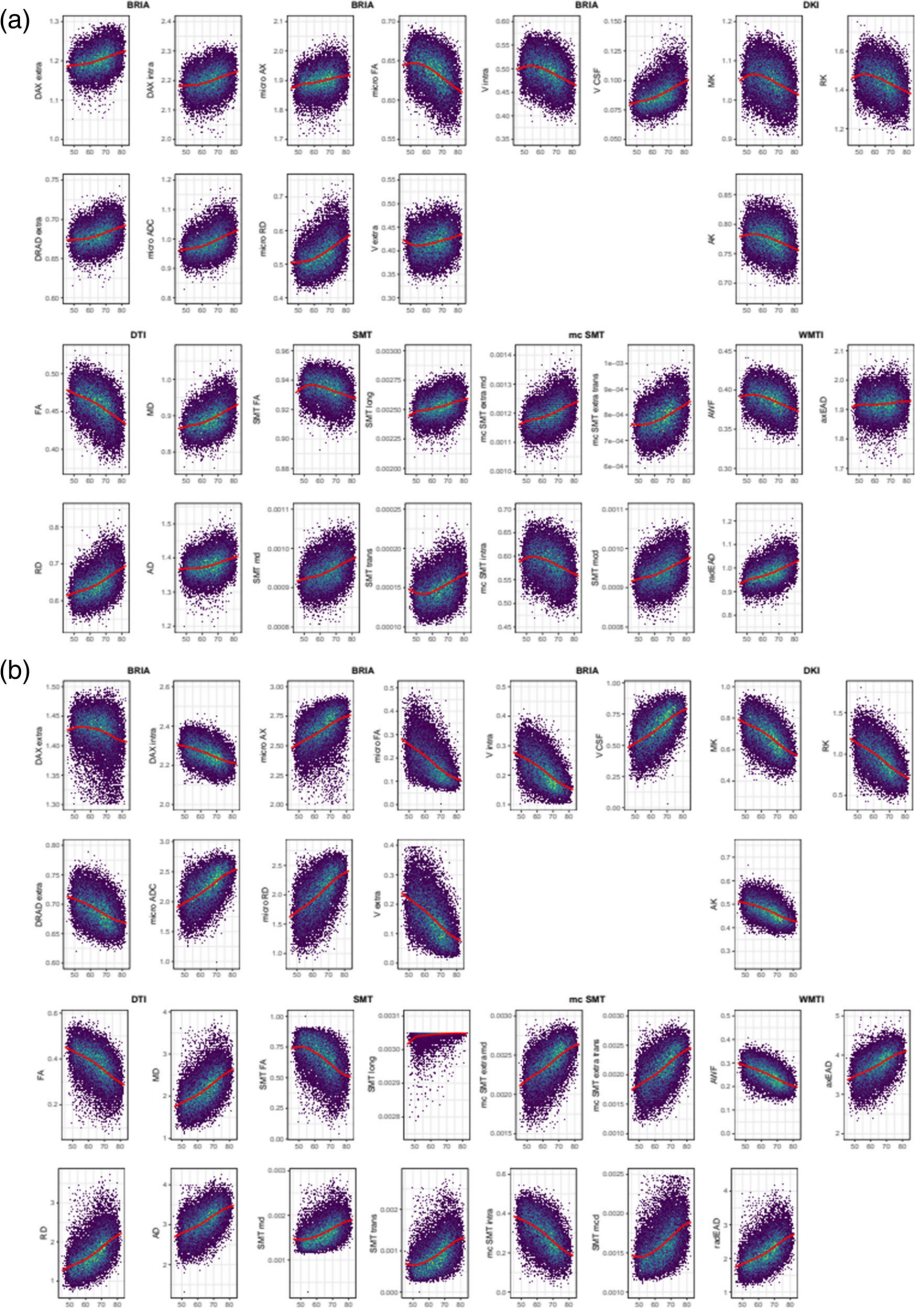
Whole‐brain and fornix diffusion metrics across age. The presented plots represent diffusion metrics for each of the six diffusion models from the full sample *N* = 35,749 for (a) whole‐brain diffusion metrics, (b) fornix diffusion metrics. Brighter colors indicate higher density and red lines are fitted lines to the relationship between age and diffusion metric. Plots for forceps can be found in Figure S14.

In Figure 9 we present absolute diffusion metrics for the whole brain (Figure 9a) and fornix (Figure 9b) across ages for the examined six diffusion approaches (for forceps see Figure S14; overview of metrics: Table S10). Age‐metric relationships for fornix were approximating linearity closer than more curvilinear global age‐curves.

Several fornix‐age relationships for BRIA extra‐axonal and intra‐axonal radial and axonal diffusivity opposed age relationships of whole‐brain‐averages, whereas forceps‐age relationships closely resembled these whole‐brain‐average metrics' age relationships.

**FIGURE 10:**
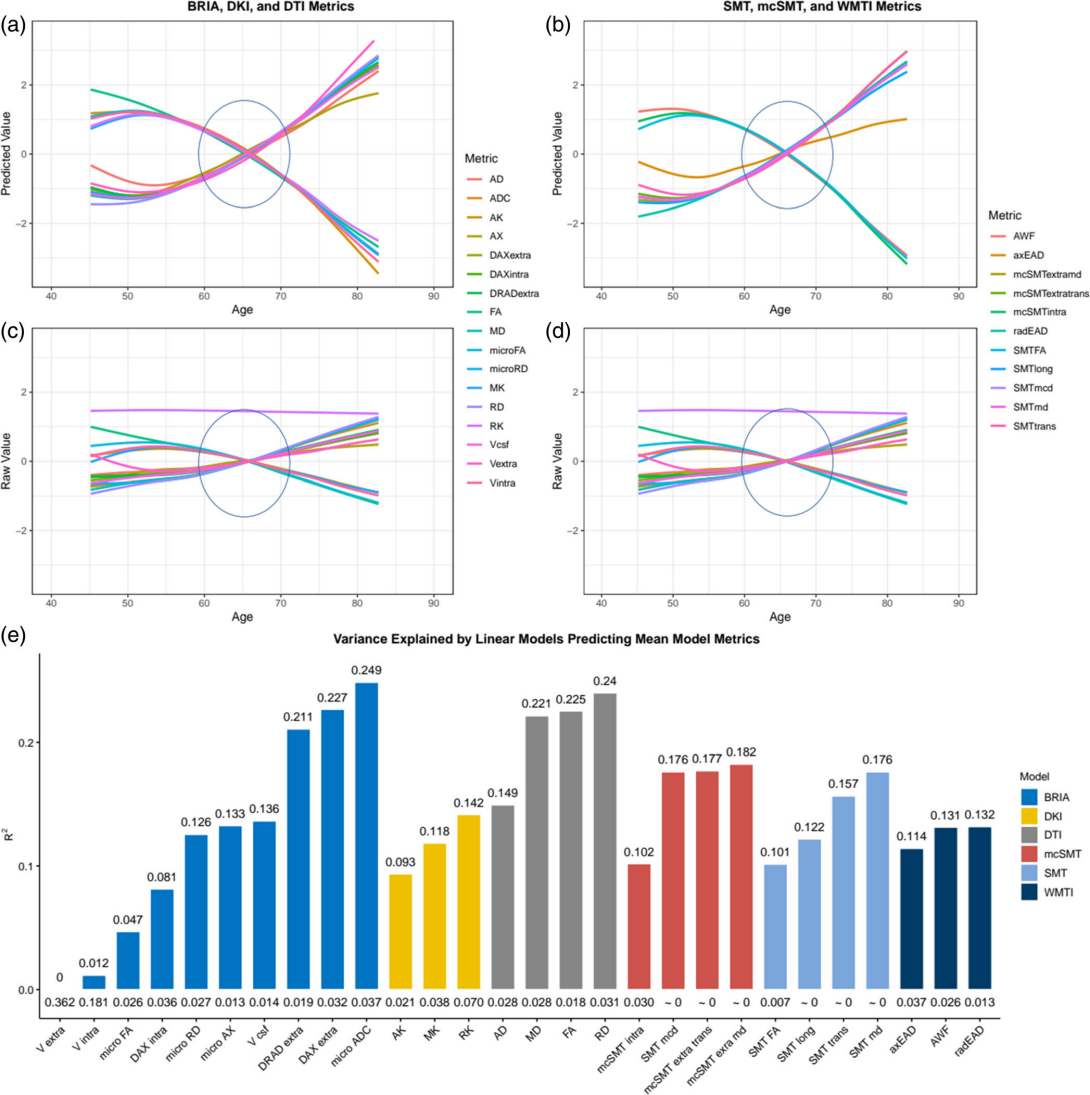
Raw and predicted whole‐brain WM diffusion metrics by chronological age. Figure 10a–d shows age curves for each standardized (z‐score) diffusion metric's mean skeleton value (y‐axis) plotted as a function of age (x‐axis). Shaded areas represent 95% CI. Curves fitted to raw values (Figure 10c,d) serve as a comparison to the lm‐derived predicted values from Equation (4) (Figure a,b). Figure 10e indicates the model fit for the linear models from Figure 10a,b, showing *R* ^2^adj values on top and standard error (SE) on the bottom of the bars which each represent a Fornix skeleton value for one of the seven models. Lines crossing at age 65 are marked with ovals. Model summaries of all 28 mean models can be found in Table S5. The same visualization of fornix diffusion values can be found in Figure S9, and for the forceps minor in Figure S15.

Whole‐brain (Figure 10), fornix (Figure S9), and forceps (Figure S15) diffusion metrics M were predicted from age, sex and scanner site to create age curves (Figure 10a,b) which can be compared to crude curves (Figure 10c,d). Highest SE, *R* ^2^adj, and variability across metrics was observed when predicting BRIA metrics (*R* ^2^adj = 0.21), as well as lowest *R* ^2^adj ≈ 0 in BRIA Vextra, respectively. While DTI metrics could also be predicted well from the model, lowest variability in *R* ^2^adj was found in WMTI and DKI. For fornix metrics, SE and *R* ^2^adj was generally higher across diffusion approaches (Figure S9).

Likelihood Ratio tests indicated age dependence across global metrics (*p*Holm < .001), with the exception of WMTI axEAD (χ^2^ = 6.66, *p*Holm = .084; Table S11), whereas all fornix (Table S4) and forceps (Table S17) features were age sensitive. While the regression lines show a slight curvature, model fit did not differ between linear and nonlinear models for whole‐brain (Table S12), fornix metrics (Table S9), and forceps minor metrics (Table S18), indicating steady WM degeneration in mid‐life to older ages.

### 3.5 Associations between BAG and WM

Finally, principal components of regional and whole‐brain WM metrics for each of the eight models (Table 2) were only weakly correlated with uncorrected BAGu, and similarly related to corrected BAGc, chronological and predicted ages (Figure S10). Furthermore, when predicting either WM components which explain most variability (Figure S10, Table S14) or single regional or whole‐brain metrics (Figure S11) from BAGc and BAGu and covariates, models predicted relatively small proportions of variance, with small contributions of BAG to the model (Figures S10, S11).

## 4 DISCUSSION

We revealed that both conventional DTI and advanced diffusion approaches (WMTI, DKI, BRIA, SMT, mcSMT) perform consistently on brain age predictions, as indicated previously (Beck et al., 2021). As a novel finding, our results show strong contributions of fornix and forceps minor microstructures to brain age prediction models. Additionally, among WM features, fornix shows strongest correlations with age. This suggest that the fornix and forceps minor are key WM region of cross‐sectional brain age, with fornix and whole‐brain dMRI metrics' age trajectories following similar patterns such as steepening slopes at later ages. Furthermore, WM microstructure is expected to steadily degenerate in midlife to older ages, in particular, in extra axonal space.

### 4.1 Limitations

There are multiple challenges related to fornix and forceps minor as drivers of brain age estimates, particularly multicollinearity, which might bias estimates of the importance of fornix and forceps minor (gain and permutation feature importance) for brain age predictions, and second, data processing artefacts. UKB offers diffusion data acquired with the most typical two‐shell‐diffusion protocol. Nevertheless, the standard diffusion model (Novikov et al., 2018) based on differentiation of intra‐ and extra‐axonal water pools could not be solved using this measurement strategy (Novikov et al., 2018). As a result, the derived diffusion metrics have both numerical uncertainties and the variability introduced from nonbiological parameters (Novikov et al., 2018). Quantitative metrics derived from the different diffusion approaches allow to investigate such nonbiological variability and to grade the subject variability in terms of used covariances. Yet, the aforementioned technical limitation might play a decisive role in a clinical context (Novikov et al., 2018; Thomas et al., 2011).

Besides obstacles resulting from modelling assumptions, our sample is cross‐sectional in design and limited to adults older than 40, which, in turn, influences predictions (de Lange et al., 2022). Additionally, the UKB imaging subsample shows better health than the non‐imaging UKB subjects (Lyall et al., 2022). Another open question is the exact interpretation of BAG and its relationship with WM metrics. This BAG‐WM relationship was found to be small for principal WM components (Figure S10) and single diffusion metrics (Figure S11). Previous research indicates no relationship between the rate of change in longitudinal regional and global T1‐weighted‐feature‐retrieved BAG (Vidal‐Pineiro et al., 2021). Yet, further investigation of longitudinal, in particular voxel‐wise WM‐derived BAG provides additional avenues to increase the interpretability of BAG.

Diffusion metrics were highly correlated within fornix (Figure 7) and forceps (Figure S13) across diffusion approaches, and show similar age trajectories (fornix: Figure S9, forceps: Figure S15). This provokes the question of redundancy of some of the metrics. The identification of redundant metrics and the combination of metrics across diffusion approaches is a matter of future research comparing diffusion approaches by probing them in practical settings such as in clinical samples (Kantarci, 2014).

Only few studies (Chen et al., 2015; Christiansen et al., 2016) address the fornix across ages. A possible reason is fornix’ artefact‐susceptibility induced from its proximity to the cerebrospinal‐fluid, while being a small tubular region. Recent processing pipelines such as TBSS minimize such artefacts (Smith et al., 2006). Yet, the influence of cerebrospinal‐fluid artefacts in small tubular structures like the fornix remains unclear (Bach et al., 2014). Fornix is a relatively small anatomical structure, and, for example, fornix BRIA cerebrospinal‐fluid fraction is higher (vCSF > 0.5) than global measures (vCSF > 0.075), suggesting a presence of strong partial volume effect. In order to overcome such distorting effects, voxel‐wise techniques are recommended, demanding the development of novel approaches incorporating techniques such as deep learning showing better performance than traditional ML, especially on large population samples (Popescu et al., 2021).

### 4.2 Consistency across diffusion approaches

Overall, the results of brain age predictions are similar across diffusion approaches, with WMTI, DTI, and DKI predicting age better than SMT, mcSMT, and BRIA considering model fit and prediction‐outcome correlations (Table 2). This finding could be explained in terms of diffusion approaches, that is, the attempt to introduce more biophysically accurate parameters into the model might simultaneously reduce the general sensitivity of the used approaches to tissue changes. Integrative approaches such as DTI or DKI are able to localize brain changes, however, without providing information about the underlying mechanisms. Our study supports a previous study with a smaller but more age‐differentiated sample (*n =* 702) of DTI and WMTI being superior to mcSMT at brain age predictions in terms of model performance (Beck et al., 2021). When examining additional diffusion models on a larger sample, and also including JHU ROIs in addition to tract and whole‐brain average scores, we find DKI metrics to have higher predictive power than in Beck and colleagues (Beck et al., 2021). This effect might be partly due to added spatial detail from the added RIOs and their relationships to the tracts. Simultaneously, differences between diffusion approaches, and both variance explained and prediction error (root mean squared error, mean absolute error) were smaller in this study. These differences are likely due to the narrower age range in our study (de Lange et al., 2022), whereas our significantly larger sample emphasizes the reliability of our findings.

While brain age predictions from single diffusion approaches were grossly similar, predictions from combined approaches were most accurate (Table 2). Correlations between predicted and chronological age were consistent across diffusion approaches, as differences between correlations were small (Figure 5, Figure S8). This shows that addressing a wider range of WM characteristics improves predictive models compared to models with single diffusion approach metrics (e.g., only DTI), which would be intuitive when considering BAG as a general indicator of health (Beck et al., 2022; Cole et al., 2017; Kaufmann et al., 2019; Leonardsen et al., 2021).Vice versa, reducing spatial specificity by averaging diffusion metrics across all WM reduced prediction accuracy. Conventionally used DTI on its own is limited in its ability to present biophysically meaningful measures of the underlying microstructure. As a result, the advanced modelling is recalled including intra‐ and extra‐axonal spaces and tissue peculiarities being influenced by individual differences in myelin and fiber architecture (crossing/bending fibers, and axonal characteristics; Beck et al., 2021). Hence, adding additional information to DTI better allow to infer the underlying neurobiology of tissue, for example, expressed in differential WM‐age‐dependences (Figures 9, 10, Figures S14, 15) or brain age predictions (Table 2; Beck et al., 2021).

We observed that BAG exhibits strong correlations across all diffusion approaches (Figure 6, Figure S1). Congruently with the correlational differences (Figure 5, Figure S8), BAG based on averaged skeleton values was least correlated to all other diffusion approaches (Figure 6), indicating inferiority of global compared to region‐wide approaches. BAG obtained from WMTI, DTI, and DKI were closest related to BAG from the multimodal approach (which predicted age best), both for age‐bias corrected and uncorrected BAG (Figure 6, Figure S1). This is in agreement with the observed age‐prediction model performance (Table 2). BAG correlations were observed in three clusters: (1) WMTI and DTI, (2) mcSMT, SMT, BRIA, and (3) DKI, indicative of similar measurements within these clusters (Figure 6, Figure S1). To a certain extent, these clusters reflect similarities in the underlying mathematics of the clustering diffusion approaches. For example, mcSMT and SMT are closely related models (Kaden et al., 2016a), whereas DKI's non‐Gaussianity might reveal another quality of age‐sensitive WM microstructures not captured by the other approaches (De Santis et al., 2011). Additionally, the cluster differences indicate that the observed diffusion approaches measure different age(ing)‐sensitive characteristics, supporting the argument for a combination of diffusion approaches when assessing the ageing brain.

### 4.3 Age trajectories and fornix and forceps minor as a brain age feature

Based on the presented findings on fornix, we further investigate details of fornix, keeping discussed limitations to the generalizability of the findings in mind. Diffusion metrics describing fornix microstructure were consistently related to each other and age across all diffusion approaches in two clusters. Values were positively correlated within each cluster and negatively between clusters (see Figure 7). In the first cluster, different approaches' FA, kurtosis metrics (MK, RK, AK), water fractions (vintra and vextra from BRIA and AWF from WMTI), and BRIA intra‐axonal and extra‐axonal radial and AD were positively correlated. The second cluster, which was negatively related to the first cluster but positive to age, contained metrics of MD, AD, and RD, and cerebrospinal‐fluid fraction of the different diffusion approaches, which were positively related to each other. Interestingly, both clusters consisted of unit‐less values, for example, water fractions, and diffusivities, which might have the same meaning as extra‐axonal ADs from different diffusion approaches, for example, BRIA versus SMTmc. Such consistencies of similar metrics across diffusion approaches were more apparent for the fornix when QC‐identified outliers were removed (compare Figure 7 and Figure S4), which supports the reliability of our findings of fornix‐age‐dependencies. Furthermore, fornix metrics were most strongly related to age across diffusion approaches (Figure 8, Figure S11), supporting the importance of fornix in reducing error of brain age predictions (Table 3). Correlations of diffusion metrics within the forceps minor were not as strong and consistent as in the fornix, and partly in the opposite direction as for the fornix (Figure S13). Not surprisingly, all fornix and forceps minor features were age‐sensitive (Tables S4, S17), and more age sensitive than whole‐brain metrics (compare: Table S11). Whole‐brain trajectories are in agreement with previous results, showing‐age sensitivity of various mean diffusion metrics (Beck et al., 2021), and the same directionality of age trajectories of metrics for DTI (Cox et al., 2016a; Westlye et al., 2010), mcSMT, DKI, WMTI (Beck et al., 2021).

We displayed differential behaviors of fornix microstructure measures across diffusion approaches (Figures 9, 10). Focusing on absolute diffusion values (Figure 9), diffusion measures which are correlated (Figures 6, 7, Figure S13) exhibit similar age dependences. Additionally, slopes of fornix compared to whole‐brain diffusion metrics were generally steeper and closer approximating linearity, indicating stronger changes, such as quicker WM degeneration in the fornix compared to the whole‐brain average (see Figure 9). Particularly BRIA metrics show visually detectable differences between the fornix and the whole brain (Figure 9, DAXextra, DAXintra, DRADextra, Vextra); as opposed to global age trends which are also strongly resembled by forceps minor (Figure S14), fornix intra and extra‐axonal diffusion decreased, indicating fornix shrinkage with increasing age. Periventricular shrinkage is linked to enlarging ventricles (Kwon et al., 2014), which has been related to ageing and neurodegenerative disorder progression (Pinaya et al., 2021). This effect was observed by a positive relationship between age and cerebrospinal fluid (CSF) fraction in BRIA. Another metric which revealed larger differences in the fornix than for the whole‐brain average was intra‐axonal water fractions, which can be treated as a proxy for the axonal density, decreased with increasing age (see Figure 9, BRIA:Vintra; SMTmc:intra; WMTI:AWF) while the CSF fraction (BRIA) increases. Such WM microstructure changes are not only directly linked to different neurobiological features but can be markers of clinical outcomes, such as dementia (Meeter et al., 2017; Thomas et al., 2011).

A selection of metrics is comparable across diffusion approaches when taking DTI as reference point and focusing on similar age trends. DTI metrics AD, RD, and MD tend to increase over the lifespan and FA tends to decrease across brain regions (Figures 9, 10; Beck et al., 2021; Cox et al., 2016b; Davis et al., 2009; Westlye et al., 2010) as well as in fornix (Figure 9b, Figure S9), implying processes such as de‐myelination, changes in axonal and general WM integrity. Such DTI‐age‐dependencies are reflected by according BRIA, SMT, and WMTI metrics, whereas DKI shows opposite age‐relationships, as presented previously (Beck et al., 2021). Deterioration effects, measured by the age‐dependency of axonal water fractions, were generally stronger in fornix compared to whole‐brain metrics (Figure 9). Interestingly, opposed to global metrics, radial diffusivity measures from DKI and BRIA (DRADextra) decreased in fornix (Figure 9), suggesting higher fornix than global plasticity, potentially being an antecedent of age‐related hippocampal changes (Metzler‐Baddeley et al., 2019).

Additional, unique information about age dynamics was presented by standardized scores corrected for age, sex, and scanner site and crude standardized scores across ages (Figure 10, Figure S9). After corrections, most fornix metrics follow a tightly resembling near‐linear trend either increasing or decreasing by age (Figure S9a,b), as opposed to forceps minor (Figure S15) and whole‐brain metrics which follow a rather curvilinear line, as previously shown (Beck et al., 2021; Davis et al., 2009; Westlye et al., 2010). Diffusion metrics' variance explained across models indicates fornix metrics to be more sensitive to a combination of covariates age, sex, and scanner site than whole‐brain metrics (Figure 10, Figure S9). In the fornix, only BRIA extra‐axonal AD (DAX extra) and the SMT longitudinal diffusion coefficient (SMT long) showed non‐linear trajectories, however, both measures are weakly correlated to other diffusion parameters (Figure 10). Yet, when comparing model metrics such as variance explained of linear and nonlinear models predicting fornix, forceps minor, and whole‐brain diffusion metrics from age, sex, and scanner site and their interactions, there were no apparent differences between models (Tables S9, S12, S15). This implies that contrary to previous research observing the entire lifespan presenting curvilinear DTI age trajectories (Beck et al., 2021; Westlye et al., 2010), or trends toward curvilinearity (with yet better linear fit for selected regions; Davis et al., 2009), we found that fornix and whole‐brain age trajectories from age 40 can be described as linear when accounting for covariates sex, age, and scanner site. While the crossing of the x‐axis at age 65 (Figure 10, Figures S9, S15) is a reflection of the sample's age distribution (Figure 1), in addition to the shapes of the different age‐trajectories, it reveals that the different diffusion approaches are similarly age‐sensitive or measure similar underlying ageing‐related changes. For whole‐brain metrics, changes become exacerbated from 65 onward (Figure 1), with reasons potentially laying in an accelerated neurodegeneration also reflected in the exponentially increasing risk to develop neurodegenerative disorders from age 65 onward (Nichols et al., 2022). For example, in the USA, 3% of 65–74 year olds, 17% of the 75–84 year olds, and 32% of those aged 85+ developed Alzheimer's dementia (Alzheimer's Association, 2020). Subclinical or preclinical states are, however, not captured by these approximations, and WM changes usually precede clinical detections. This makes WM monitoring a promising tool for early neurodegenerative disease detection.

Beyond WM, fornix changes seem to play an important role for GM changes, particularly in the hippocampus: for example, fornix glia damages lead to hippocampal GM atrophy (Metzler‐Baddeley et al., 2019). This might be reflected by dis‐connectivity of fornix with other brain regions as described by decreasing extra axonal space coefficients (Figure 8b), and following changes in fornix function. Potentially, the consequences of age‐related fornix changes thereby affect functionality of a selection of brain regions, such as the hippocampus. While several studies have presented ageing‐related fornix microstructure changes in humans (Chen et al., 2015; Christiansen et al., 2016) and monkeys (Peters et al., 2010) in small samples, only one large‐scale study revealed findings connected to the fornix, namely strongest default mode network GM volume covariation with fornix WM microstructure (Kernbach et al., 2018). This suggests that fornix, a key connector of the limbic system with the cortex, might also be critical for default mode network functioning. Moreover, memory and episodic recall have been related to fornix (Senova et al., 2020). Hence, fornix changes might play an important role in known ageing‐dependent temporal lobe changes, and specifically hippocampal changes for ageing‐related pathological developments (Cabeza et al., 2018; Burke & Barnes, 2006; Hedden & Gabrieli, 2004; Pluvinage & Wyss‐Coray, 2020). Previous studies presented age‐related fornix DTI metric changes (Chen et al., 2015; Christiansen et al., 2016; Metzler‐Baddeley et al., 2019) which potentially appear prior to hippocampal volume changes (Chen et al., 2015; Metzler‐Baddeley et al., 2019), and are related to declining episodic memory performance (Metzler‐Baddeley et al., 2019). Hence, fornix changes potentially serve to predict future pathological development, suggesting fornix WM microstructure and changes in such as ageing biomarkers. This supports previous findings showing network re‐activations, metabolic, and GM changes after fornix deep‐brain‐stimulation, antagonizing the progression of neurodegenerative disorders (Jakobs et al., 2020).

Different studies showed age‐related deterioration effects in the forceps minor (Bastin et al., 2008; Fan et al., 2019), a subregion of the corpus callosum. Loss in WM integrity have also been associated with various phenotypes, for example, behavioral impacts, such as mental slowing (Jokinen et al., 2007), and various disorders, such as major depressive disorder (Won et al., 2016), schizophrenia (Kelly et al., 2018), dependencies on cocaine (Moeller et al., 2005) and alcohol (Pfefferbaum & Sullivan, 2005), with WM degeneracy explaining higher impulsivity in cocaine addiction (Moeller et al., 2005). Overall, the forceps are assumed to have an important role of connecting both hemispheres, which might be crucial for interhemispheric signal propagation (Voineskos et al., 2010). Previous research shows also that WM changes in FA and MD relate to GM thinning with the forceps being particularly vulnerable to such changes (Storsve et al., 2016). Moreover, cognitive test scores were related to forceps minor AD and MD scores in Alzheimer's Disease patients (Tu et al., 2017), and already at mild cognitive impaired forceps minor FA and MD scores were different from age‐matched participants with subjective cognitive decline (Luo et al., 2020). FA was also shown in this study as important brain age feature for both multimodal and DTI models (Table 3). This suggests forceps as an important region for brain age and ageing.

The current study gives for the first time a detailed account on region‐wise‐to‐global WM‐age relationships for multiple diffusion approaches in a representative sample, and highlights fornix and forceps minor as an important structures for age predictions across diffusion approaches. Brain age was estimated best when combining diffusion approaches, showing different aspects of WM to contribute to brain age with fornix and forceps minor being the central regions for these predictions. Trained models are made available for further research to extend the reported brain age predictions to other samples (e.g., to clinical samples with a similar age structure), in addition to examining the discussed metrics in fornix and forceps.

## AUTHOR CONTRIBUTIONS

Max Korbmacher: Study design, Software, Formal analysis, Visualizations, Project administration, Writing—original draft, Writing—review & editing. Ann Marie de Lange: Software, Writing—review & editing. Dennis van der Meer: Software, Writing – review & editing. Arvid Lundervold: Writing—review & editing, Funding acquisition. Eli Eikefjord: Writing—review & editing. Dani Beck: Writing—review & editing. Ole A. Andreassen: Writing—review & editing, Funding acquisition. Lars T. Westlye: Writing—review & editing, Funding acquisition. Ivan I. Maximov: Supervision, Study design, Data preprocessing and quality control, Writing—review & editing, Funding acquisition.

## CONFLICT OF INTEREST STATEMENT

OOA has received a speaker's honorarium from Lundbeck and is a cosultant to Coretechs.ai.

## Supporting information


**Data S1:** Supporting information.

**Data S2:** Supporting information.

## Data Availability

All raw data are available from the UKB5 (www.ukbiobank.ac.uk). Synthetic datasets with the synthpop 2016 *R* package based on the original data for all six diffusion approaches (resulting in six datasets) to run the code and code needed to run brain age predictions in Python are openly available at the Open Science Framework: (https://osf.io/nv8ea/). Synthetic datasets are simulated datasets closely mimicking the statistical characteristics of the original data while protecting data privacy and anonymity. Finally, also the trained XG Boost models are made available in the same depository.
