# Supplementary material for: Brain‐wide associations between white matter and age highlight the role of fornix microstructure in brain ageing
Source: Hum Brain Mapp. 2023 May 17;44(10):4101–19. doi: 10.1002/hbm.26333 (PMC10258541; doi:10.1002/hbm.26333)
Supplement: Supplementary file 1 — Data S1: Supporting information. [file HBM-44-4101-s001.docx]

**Supplement**

***Supplementary Figures***

**SF1: Correlations of corrected BAG and age across models**


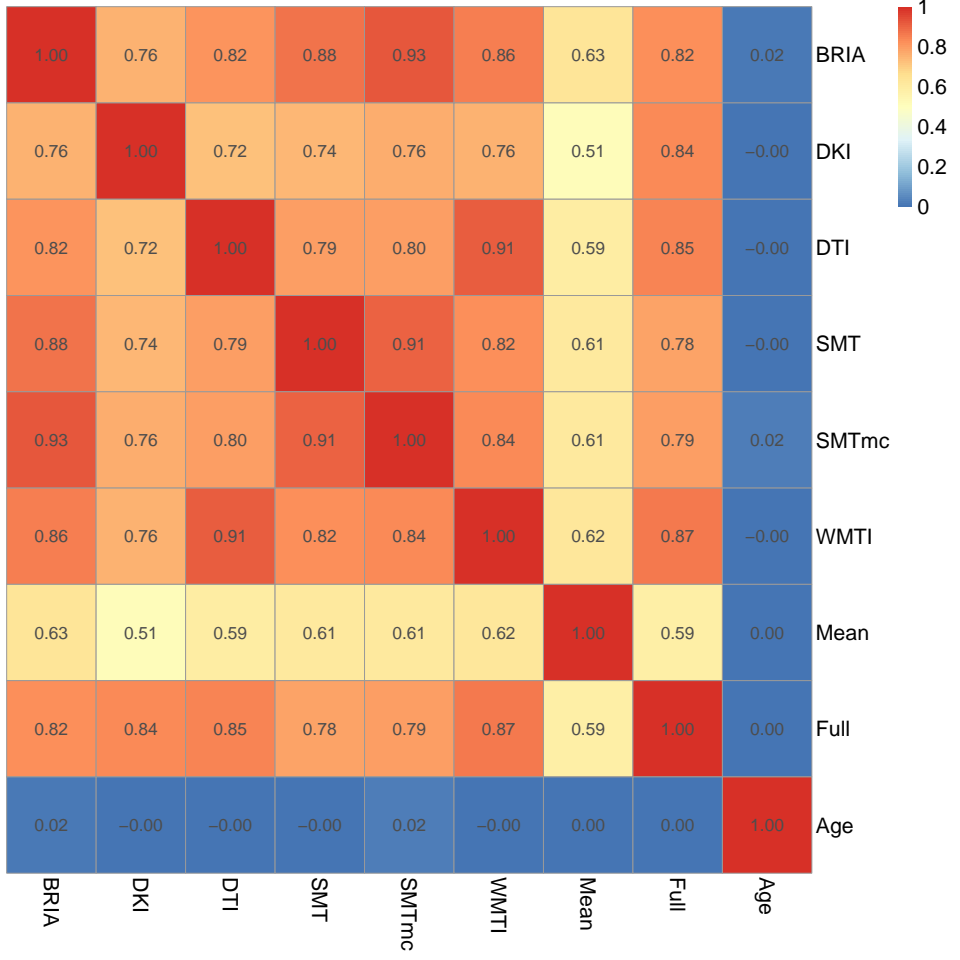
Mean = multimodal model including only mean metrics; Full = full multimodal model including all diffusion indices. Age-BAG correlations, approximating 0, were not significant at *pHolm* ≥ .05. All other correlations were significant at *pHolm* < .001.

**SF2: Comparison of predicted and raw fornix Z-scored diffusion metrics’ density**


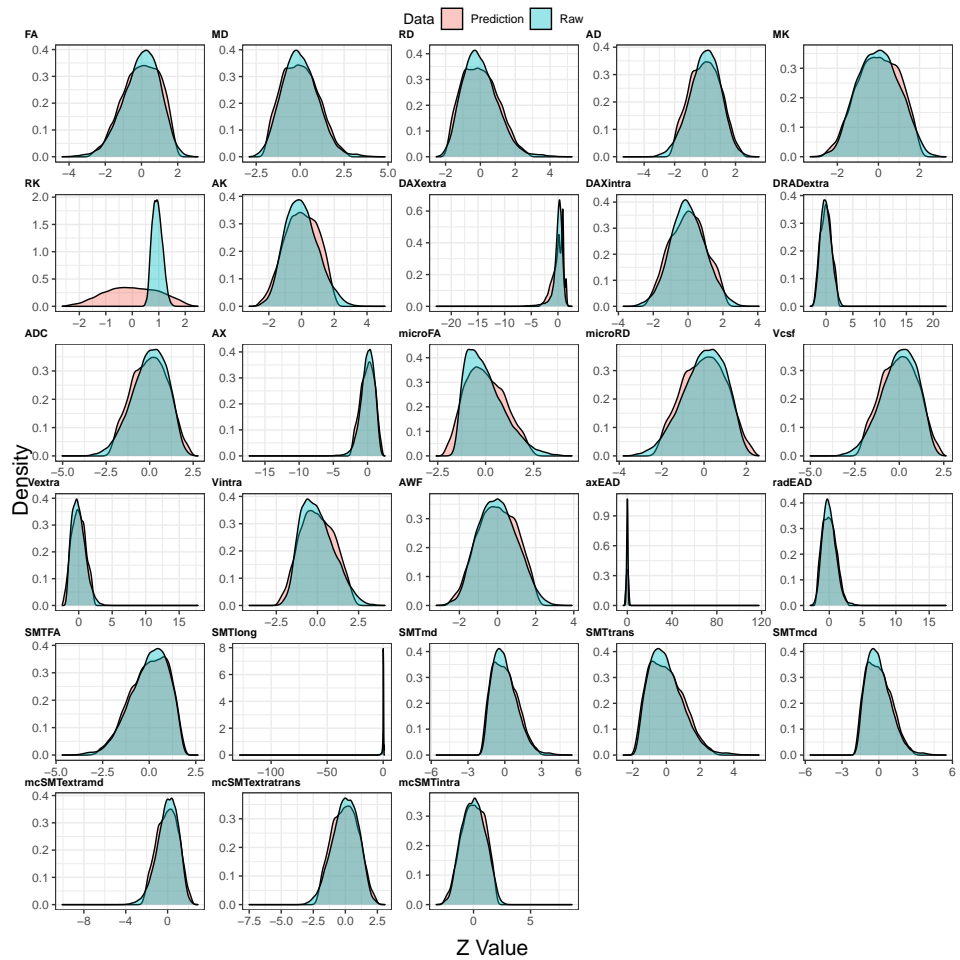
Density plots for each Z-scored (standardised) raw and predicted values for each fornix metric from the six observed diffusion models. Predictions were made from the linear model described in Equation 1.

Find the same density plot for data including QC outliers in SF3.

Supplementing the density plots, two one-sided tests for equivalence testing (TOST)87,88⁠ were used to test whether mean differences between the model’s predictions (**SF9A-B**) and the raw scores (**SF9C-D**) are equal to zero with the assumptions that observed *Z*-score differences smaller |0.5| are equal to 0. Following this assumption, differences were equal to zero for all metrics, except the DKI metric RK: Mdiff = 0.943, 95% CI [0.935, 0.951], *p* ≈ 1.

**SF3: Comparison of predicted and raw Fornix Z-scored diffusion metrics’ density including QC outliers**


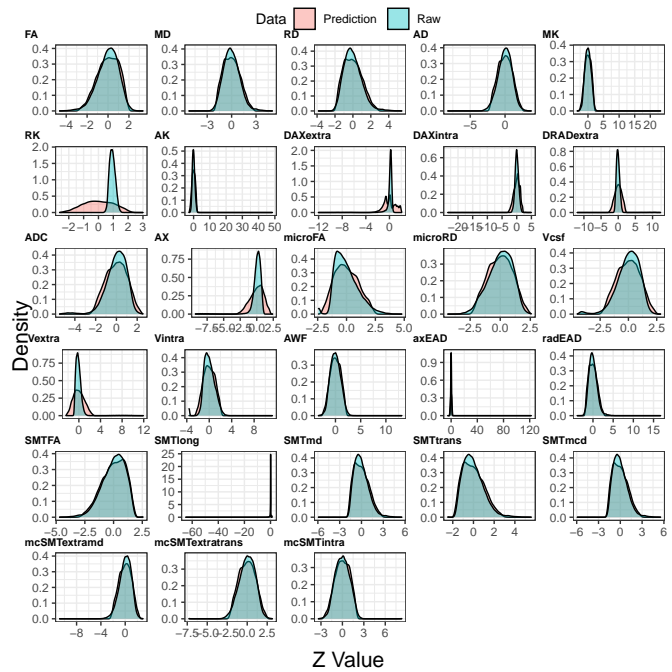


Density plots for each Z-scored (standardised) raw and predicted values for each fornix metric from the six observed diffusion models on data *containing QC outliers*. Predictions were made from the linear model described in Equation 1.

Outliers were defined by the YTTRIUM method39⁠ including outlier removal based on density-based spatial clusterisation (k-means)The total data used here was Nfull+outliers = 38,687, including the full data Nfull = 35,749 used for all analyses and Noutliers = 2,938 datasets defined as outliers. This dataset does not include participants who withdrew their consent or participants with an ICD-10 diagnosis categories G or F or stroke, category I.

**SF4: Correlations between Fornix diffusion metrics and chronological age for data including QC outliers**


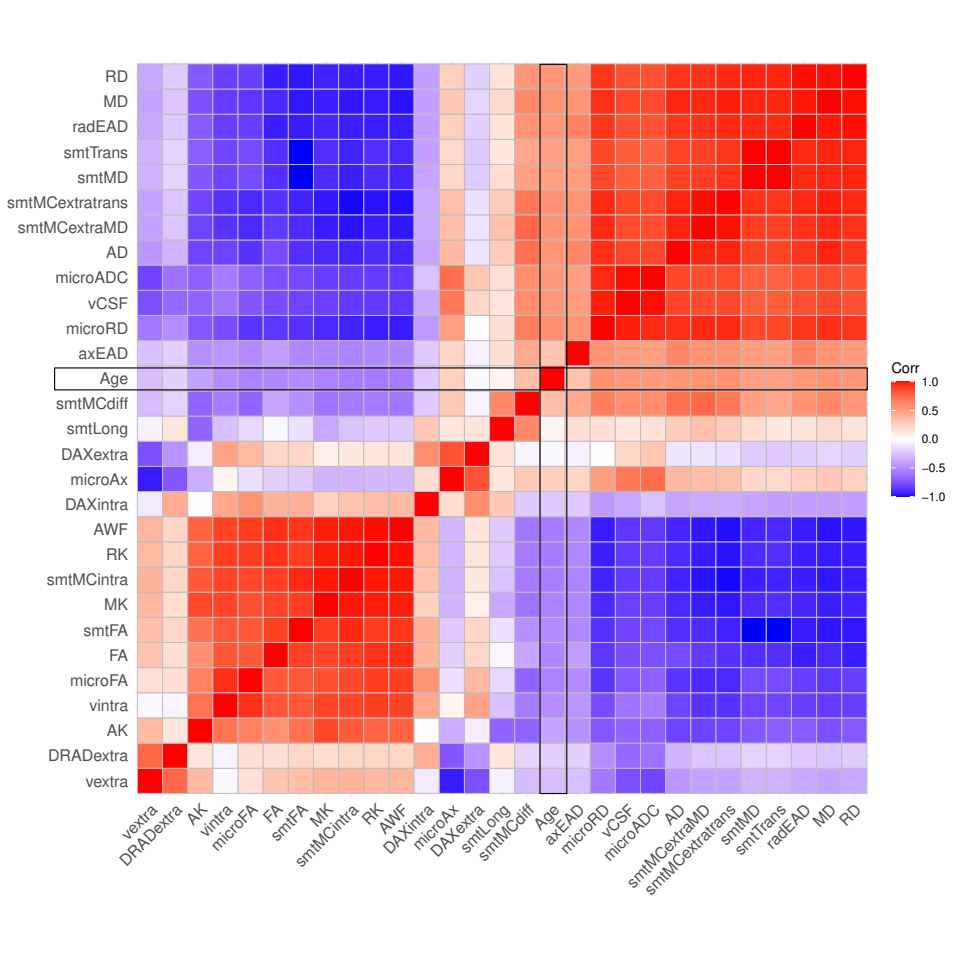
All correlations were significant at FWE-corrected *pHolm* < .05.

Outliers were defined by the YTTRIUM method38⁠ including outlier removal based on density-based spatial clusterisation (k-means). The total data used here was Nfull+outliers = 38,687, including the full data Nfull = 35,749 used for all analyses and Noutliers = 2,938 datasets defined as outliers. This dataset does not include participants who withdrew their consent or participants with an ICD-10 diagnosis categories G or F or stroke, category I.

**SF5: Density plots for the sample’s age by sex and scanner site for data including QC outliers**


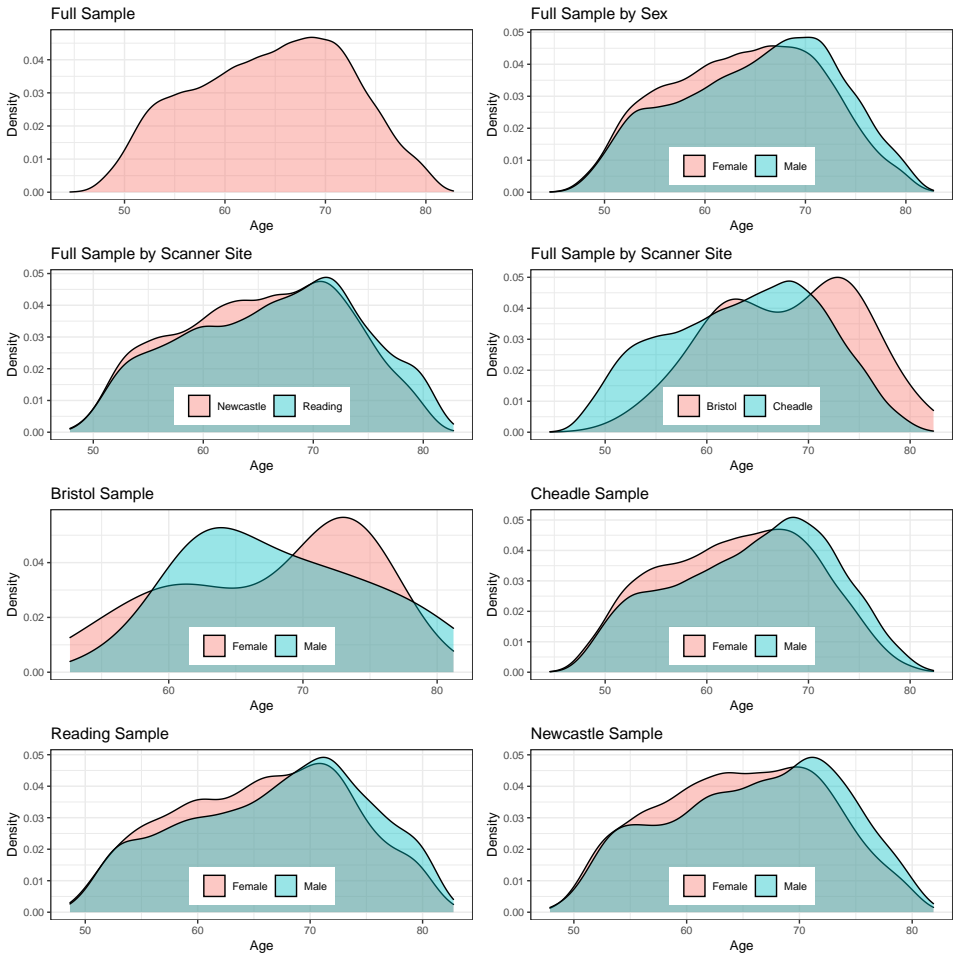
Outliers were defined by the YTTRIUM method38⁠ including outlier removal based on density-based spatial clusterisation (k-means). The total data used here was Nfull+outliers = 38,687, including the full data Nfull = 35,749 used for all analyses and Noutliers = 2,938 datasets defined as outliers. This dataset does not include participants who withdrew their consent or participants with an ICD-10 diagnosis categories G or F or stroke, category I.

**SF6: Model performance for different train-test splits for data *including QC outliers***


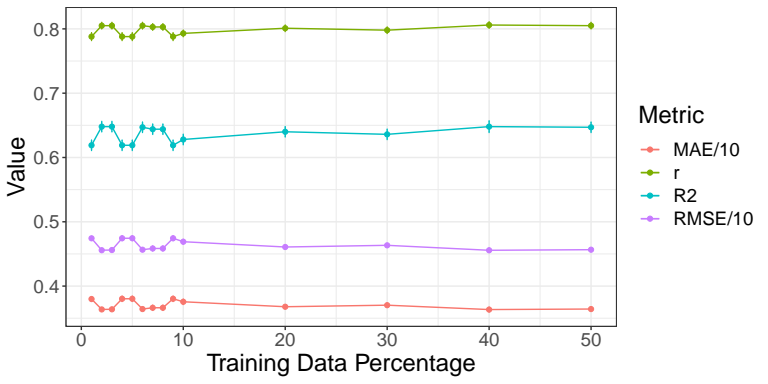
Model metrics R2, RMSE, MAE and their standard deviations, as well as the Pearson’s correlations between predicted and chronological age and its 95% confidence interval are displayed for different training data percentages of the total data (x-axis). For visualisation purposes, RMSE and MAE were divided by 10. For exact values see Suppl. Table ST8.

Outliers were defined by the YTTRIUM method38⁠ including outlier removal based on density-based spatial clusterisation (k-means). The total data used here was Nfull+outliers = 38,687, including the full data Nfull = 35,749 used for all analyses and Noutliers = 2,938 datasets defined as outliers. This dataset does not include participants who withdrew their consent or participants with an ICD-10 diagnosis categories G or F or stroke, category I.

**SF7: Correlations between diffusion metrics and chronological age for data including QC outliers**


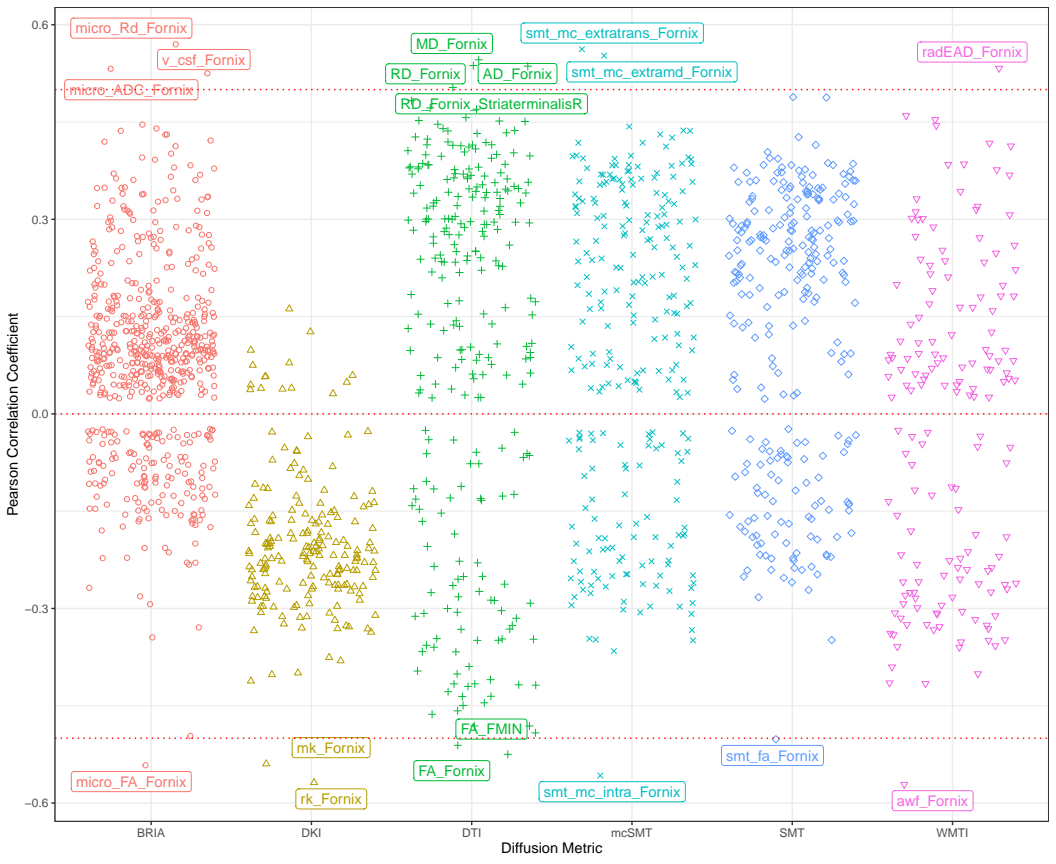
*Note*: Each point indicates one correlation between a diffusion feature and chronological age. Names of diffusion features are displayed when correlations between the feature and age reached a Pearson correlation of |*r|*>0.5. Holm correction was used for FDR-correction, and all displayed values were significant at *p* < .001.

Results for the analysis run on data *not* including QC outliers (N = 35,749) can be found in Fig.10.

Outliers were defined by the YTTRIUM method38⁠ including outlier removal based on density-based spatial clusterisation (k-means). The total data used here was Nfull+outliers = 38,687, including the full data Nfull = 35,749 used for all analyses and Noutliers = 2,938 datasets defined as outliers. This dataset does not include participants who withdrew their consent or participants with an ICD-10 diagnosis categories G or F or stroke, category I.

**SF8: Differences between correlations of chronological and *corrected* predicted age across diffusion approaches with 95% confidence interval**

**
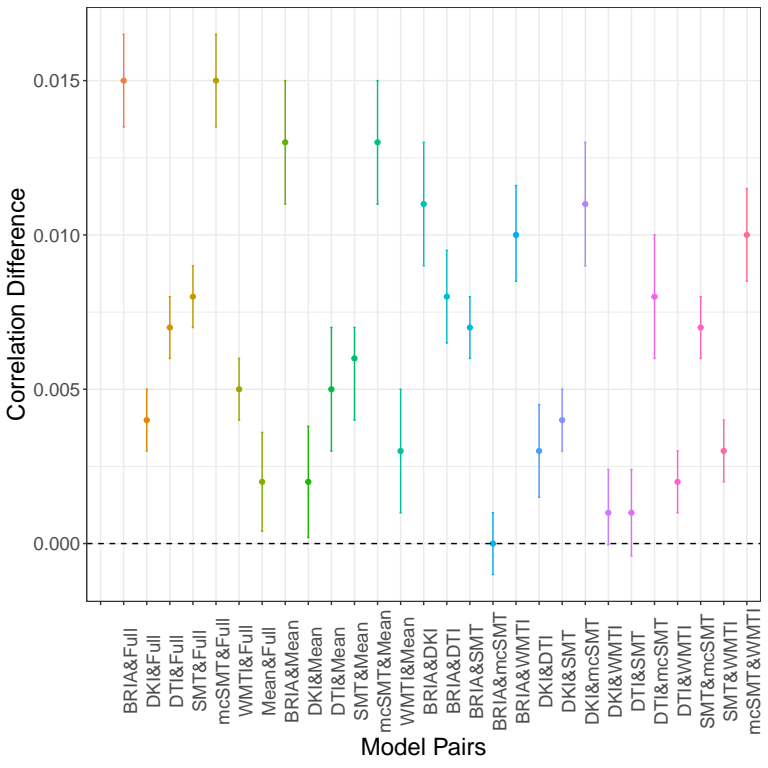
**

**SF9: Raw and predicted fornix diffusion metrics by chronological age**


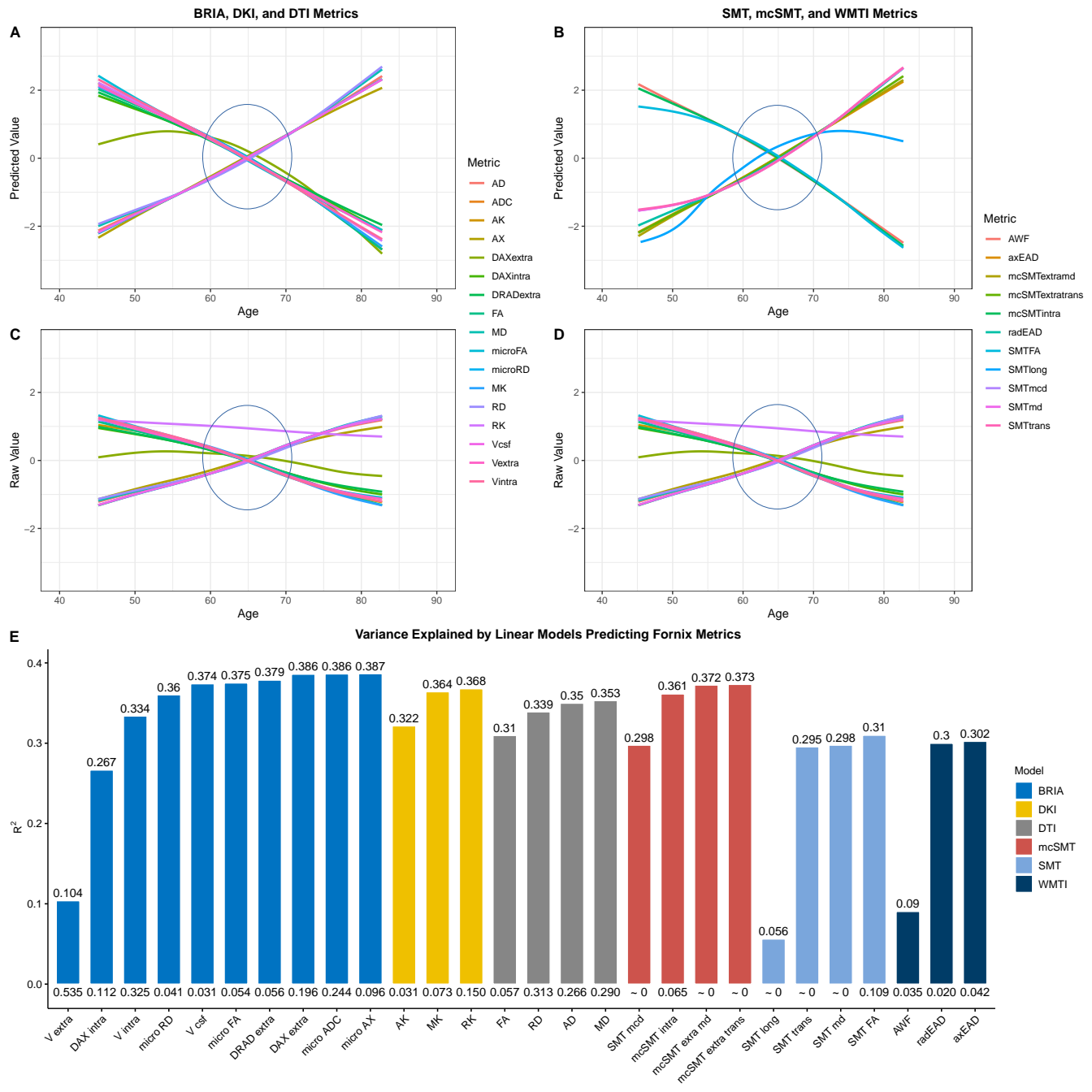
SF9A-D shows age curves for each standardised (z-score) fornix diﬀusion skeleton value (y-axis) plotted as a function of age (x-axis). Shaded areas represent 95% CI. Curves fitted to raw values (SF9C-D) serve as a comparison to the lm-derived predicted values from Equation 1 (Fig.A-B). SF9E indicates the model fit for the linear models from SF9A-B, showing R2adj values on top and Standard Error (SE) on the bottom of the bars which each represent a Fornix skeleton value for one of the seven models. Lines crossing at age 65 are marked with circles. Model summaries of all 28 Fornix models can be found in ST5. The same visualisation of diffusion values averaged across the brain can be found in Fig.10.

Model fit metrics R2adj and Standard Error (SE) for the models accounting for age, sex and scanner site (Equation 1) when predicting fornix metrics were calculated (**SF9E;** see **Fig.10** for whole brain metrics). Highest R2adj and variability across metrics were observed when predicting BRIA fornix features, lowest R2adj when predicting SMT fornix metrics. DKI, DTI and mcSMT fornix diffusion metric predictions were most consistent, with BRIA, mcSMT and SMT having one outlier each, Vextra, SMTlong, and AWF, respectively, being less sensitive to age, sex and scanner site. Highest SE could be observed in the BRIA model and the lowest SE in SMT.

To test age-sensitivity of the fornix features, likelihood ratio tests were conduced comparing models derived from Equation 1 against models derived from the same formula with age removed (Equation 2). All models showed significant age dependence, with BRIA microRD (χ2= 14,480.54, *p*Holm < .001), microADC (χ2= 14,384.87, *p*Holm < .001) and SMT vCSF (χ2= 14,311.47, *p*Holm < .001) being the most age-sensitive metrics, and mcSMT smtLong (χ2= 1,554.49, *p*Holm < .001), BRIA DAXextra (χ2= 1,824.54, *p*Holm < .001) and axEAD (χ2= 3,024.74, *p*Holm < .001) the least age-sensitive metrics (**ST4**).

**SF10. Pearson’s *r* for age, brain age and WM principal components’ relationships**

**
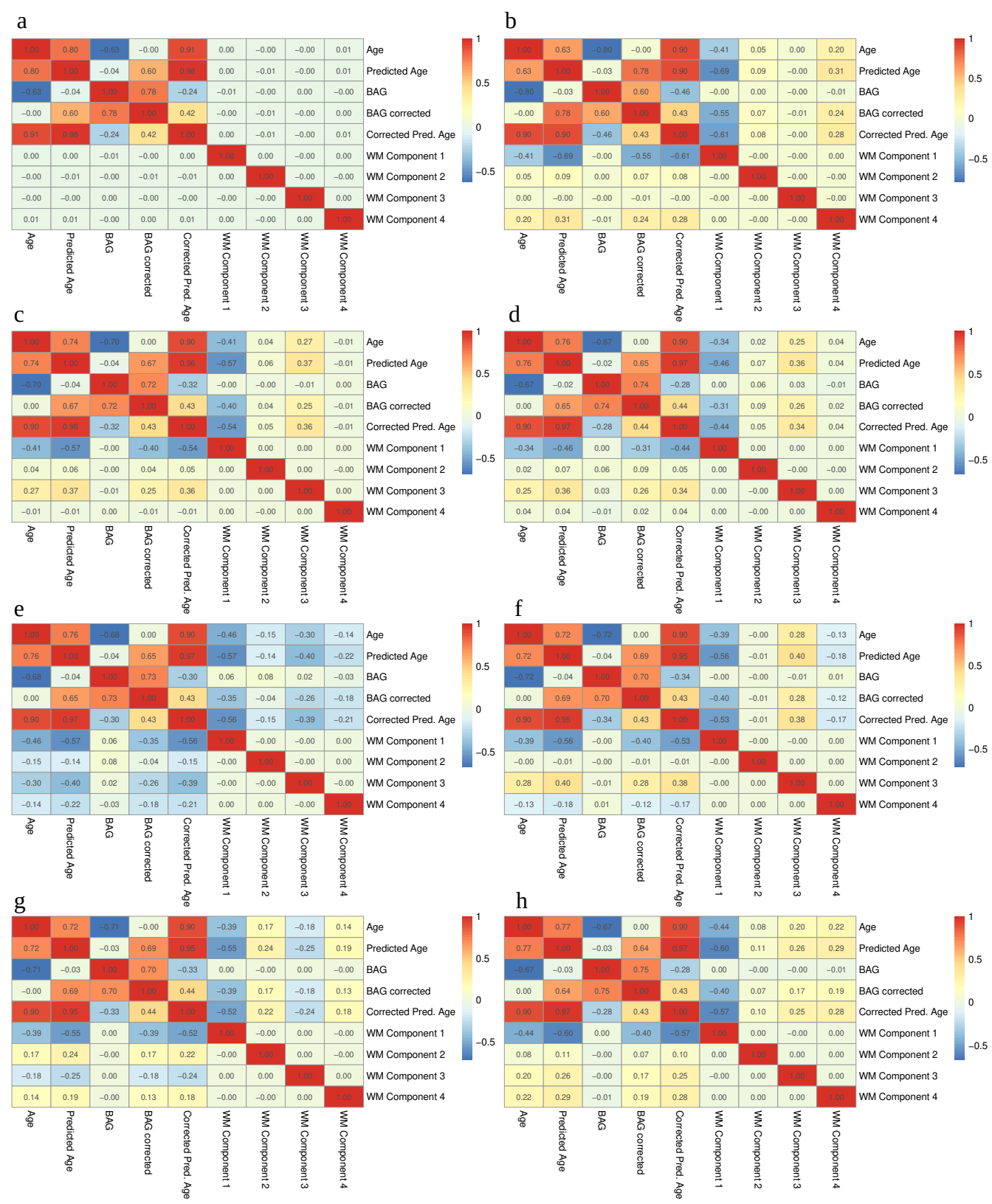
**

All correlations with Pearson’s *r* > .01 were significant at p < .001

Read row-wise from top-left to right with matrixes indicating a) full multimodal data, b) mean/whole brain average data, c) BRIA, d) DKI, e) DTI, f) SMT, g) SMT mc, h) WMTI

The first five principal components of the respective number of WM metrics for each of the eight principal components analyses were related to age, predicted (brain) age, corrected predicted age, uncorrected and corrected BAG (see **ST13** for overview of variance explained by principal components). Notably, BAG was not or only weakly related to WM components, and relationships of age, predicted age, corrected predicted age and corrected BAG with WM components followed the same pattern of direction and strength of associations, suggesting age-dependencies of these measures.

When predicting the first 4 components retrieved from the respective models (as done for brain age predictions), using BAG, age, sex, site, as well as age-sex and sex-site interactions as predictors (as specified in Equation 1), different sized proportions of the variance in the components could be explained with corrected and uncorrected BAG models not differing in variance predicted and beta values. Average data BAG models explained most variance in its first component R2 = .505, with bBAG = -0.673, followed by WMTI R2 = .372, with bBAG = -0.847, and the DTI BAG model R2 = .358, with bBAG = -1.082. The second component was best predicted by a DTI BAG model R2 = .152, with bBAG = -0.059. The third component was best predicted by the DKI BAG model R2 = .256, bBAG = 0.170, followed by the DTI BAG model R2 = .250, bBAG = -0.210; and the SMT BAG model R2 = .247, bBAG = 0.291. Finally, the last component was best predicted by the full BAG model, R2 = .128, bBAG = 0.0002. For an overview of all BAG models’ performance see **ST14**. For a more nuanced follow-up analysis of global and regional individual diffusion metric predictions see **SF11**.

**SF11.** **Predictions of individual global and regional diffusion metrics**


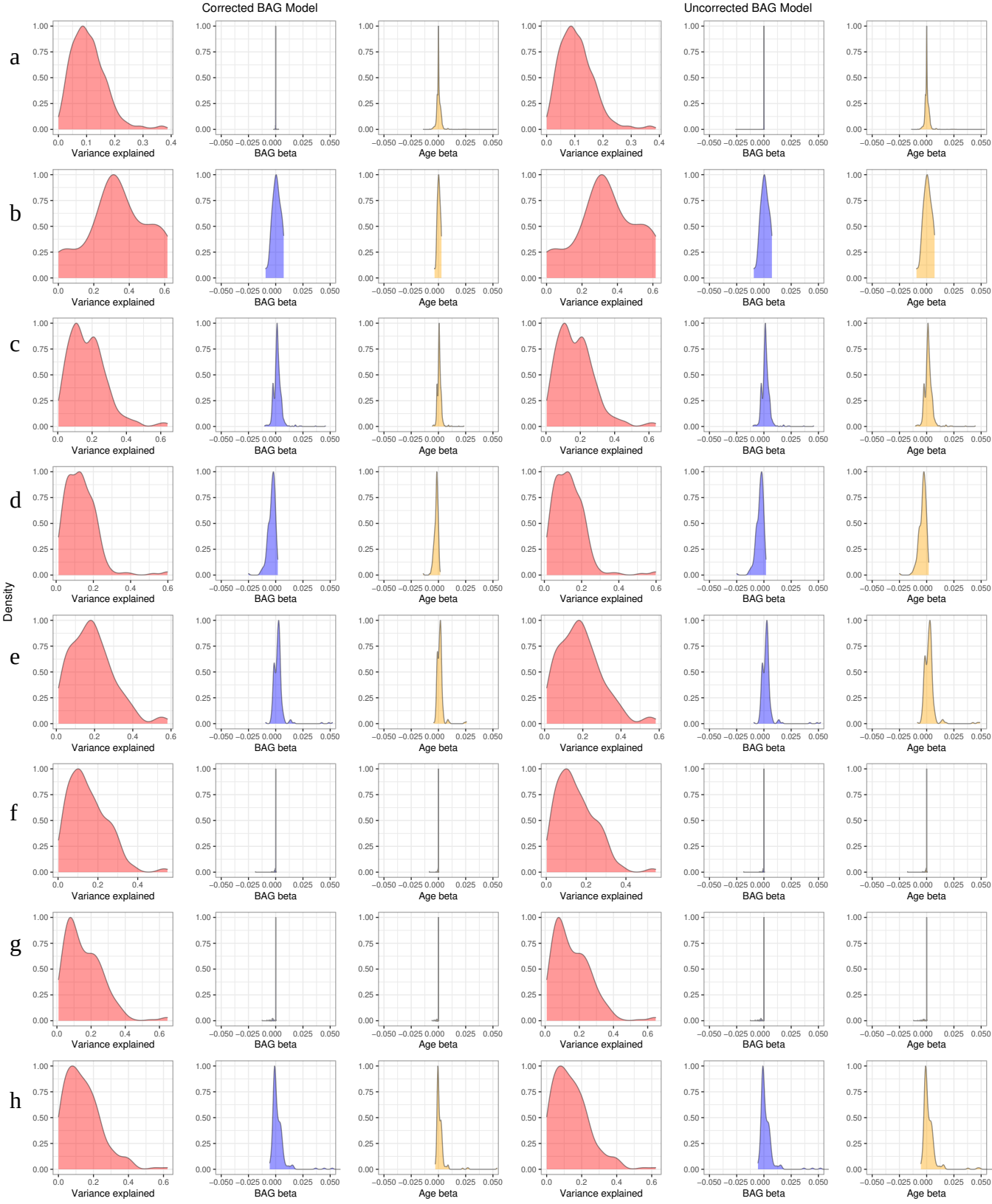


Panels indicate used models: a) full multimodal model including all approaches global and local fatures, b) mean multimodal modal, including only global metrics of all diffusion approaches, c) BRIA, d) DKI, e) DTI, f) SMT, g) mcSMT, h) WMTI.

We predicted the individual 1940 regional and global WM diffusion metrics from BAG, site, sex, age, as well as sex-age and sex-site interaction terms. While there were no differences in explaining variance between corrected and uncorrected BAG, models coefficients differed (see **SF11**).

Variance explained across statistically significant models (at Bonferroni-corrected *p* < 0.05/1940) ranged from adjusted R2min = .001 to R2max = .387 (R2mean = .108, SD = 0.062), and beta values for BAG ranged from bBAG > -0.001 to bBAG< 0.001, with most variance explained in metrics Fornix v csf (Radj2 = .387, bBAG > -0.001, bage = 0.009), Fornix micro RD (Radj2 = .386, bBAG > -0.001, bage = 0.009), and Fornix micro ADC (Radj2 = .386, bBAG > -0.001, bage = 0.019).

**SF12. Density plots feature-age correlation across diffusion approaches with tail probabilities**

**
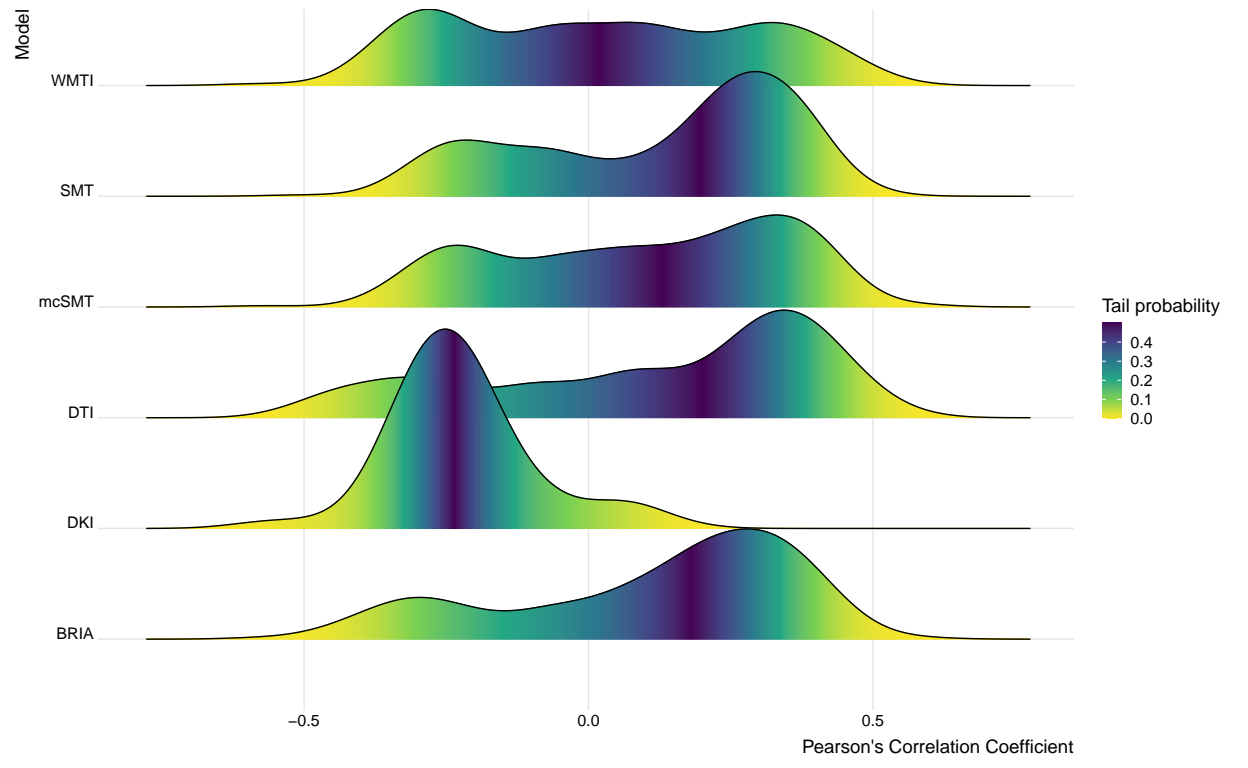
**

This figure is a supplement to Figure 5, showing the distributions of the correlations between age and each models’ diffusion metrics.

**SF13: Correlations between forceps diffusion metrics and chronological age**


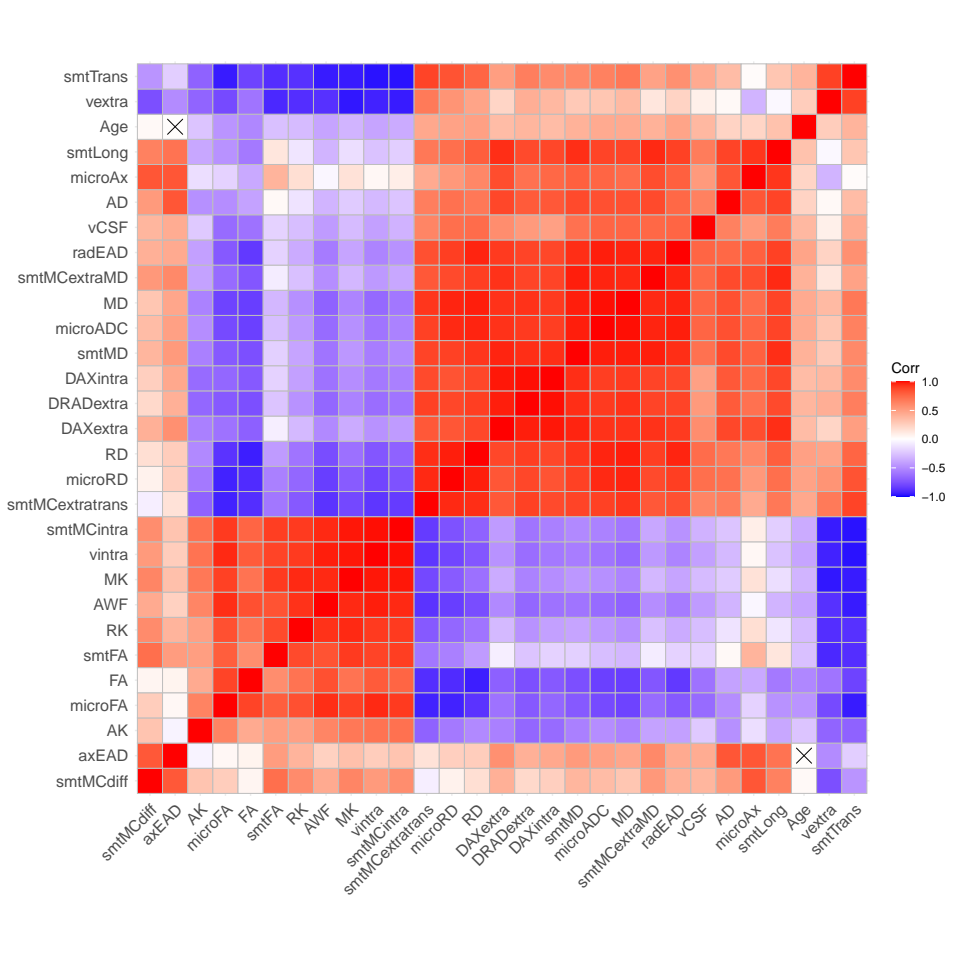
*Note*: Crossed out values were non-significant.

**SF14: Absolute forceps diffusion metrics across age**


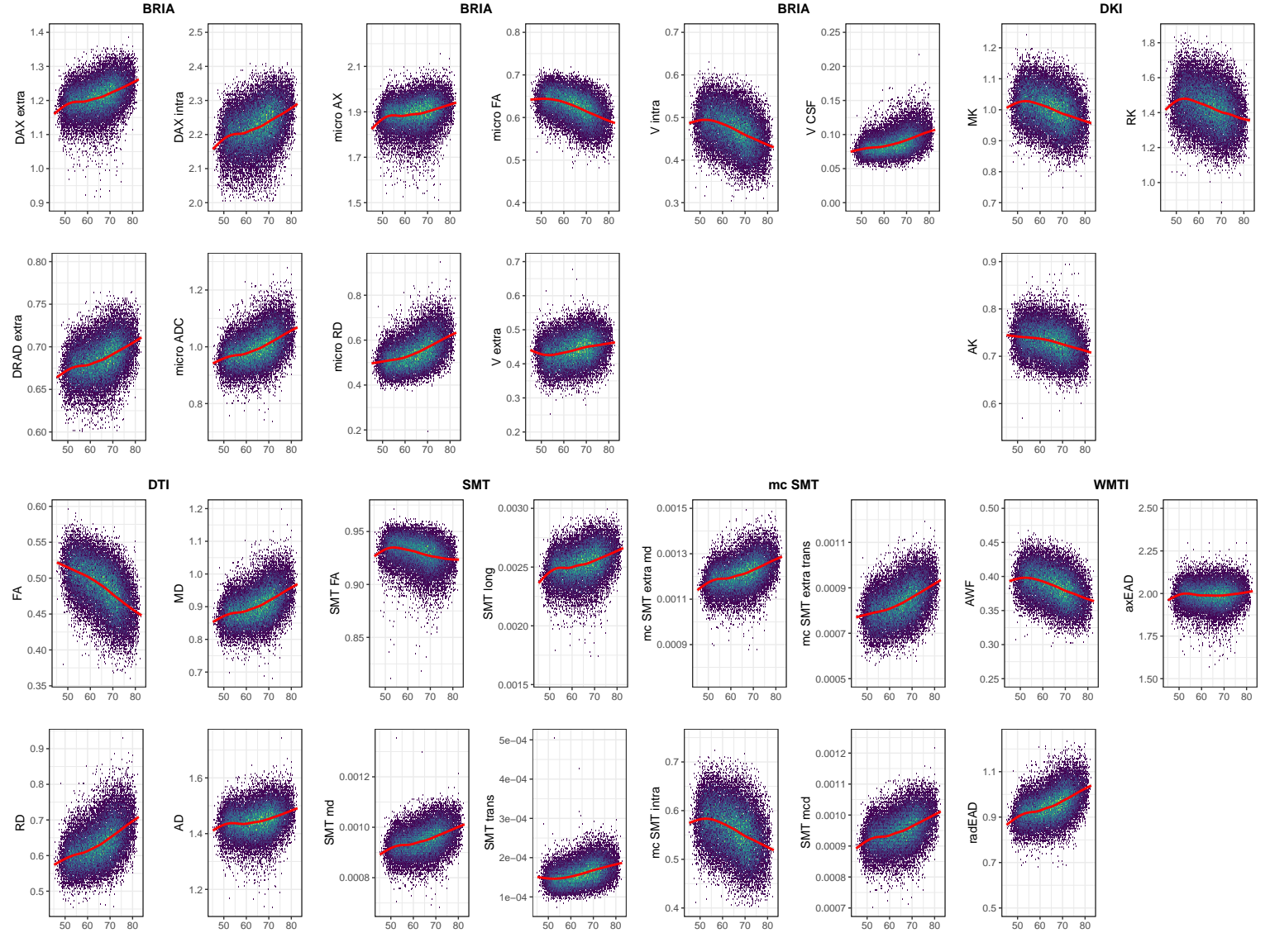
Note: The presented plots represent diffusion metrics for each of the six diffusion models from the full sample N = 35,749 for forceps. Brighter colours indicate higher density and red lines are fitted lines to the relationship between age and diffusion metric.

**SF15: Raw and predicted forceps diffusion metrics by chronological age**


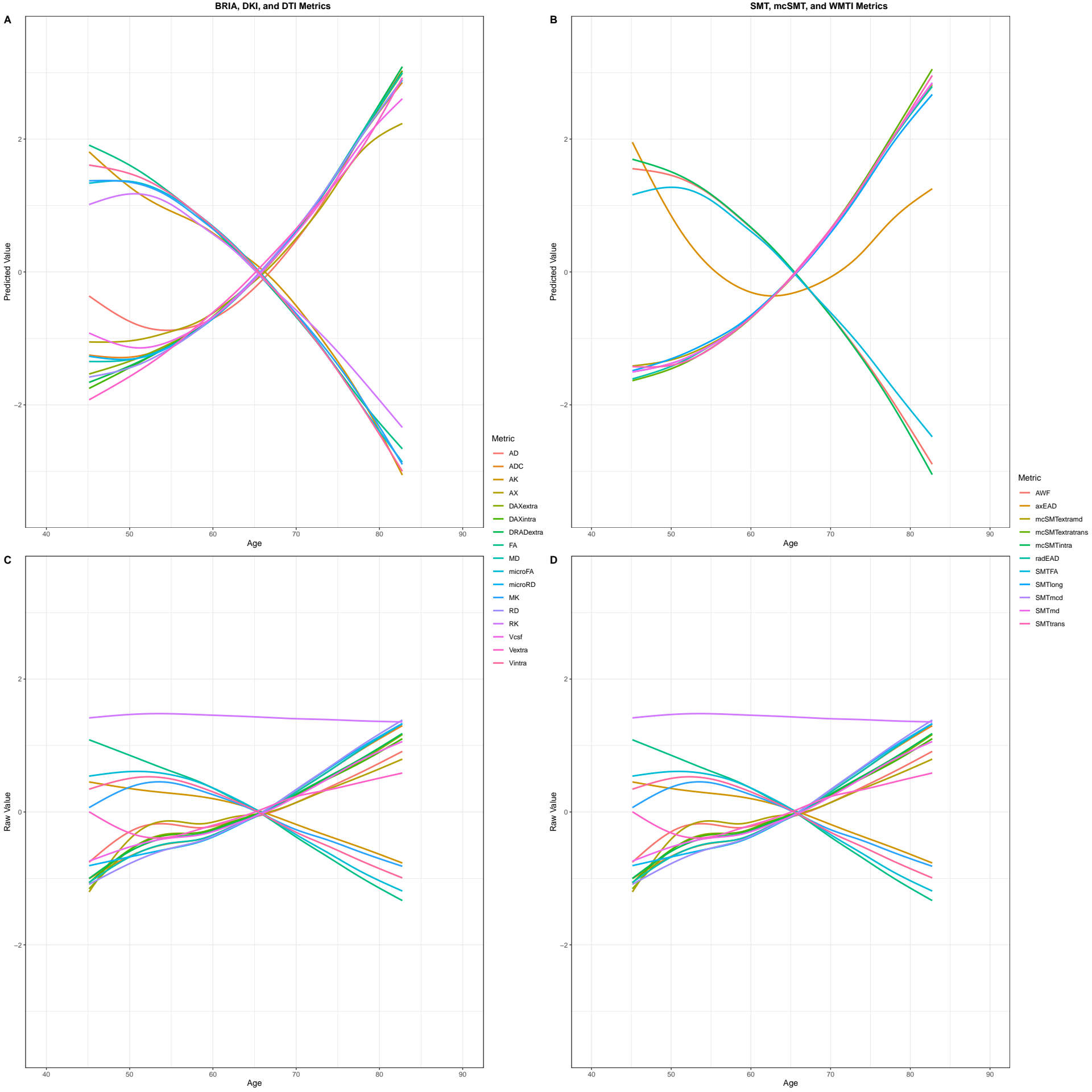


SF14A-D shows age curves for each standardised (z-score) fornix diﬀusion skeleton value (y-axis) plotted as a function of age (x-axis). Shaded areas represent 95% CI. Curves fitted to raw values (SF9C-D) serve as a comparison to the lm-derived predicted values from Equation 1.

**SF16: White Matter Tracts and Regions Used in this Study**


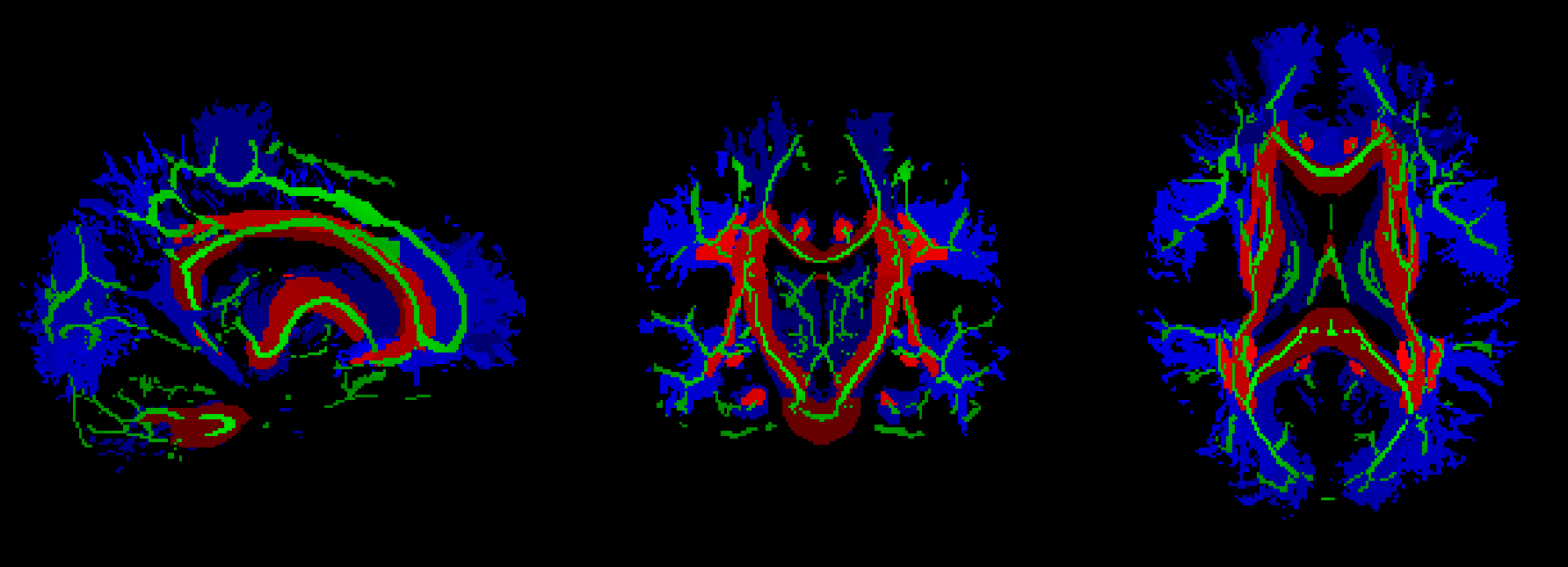
Red indicates JHU labels atlas with main ROIs. Blue indicates the tractorgaphic atlas with finer skeleton structure. The green colour marks the FA skeleton.

***Supplementary Tables***

**ST1: Brain age predictions from different train-test splits**

| **% of Data** | **Best Fitting Model** | **Train Results** | | | | **Test Results on 50% of Data** | | | |
| --- | --- | --- | --- | --- | --- | --- | --- | --- | --- |
|  |  | **R2** | **RMSE** | **MAE** | **rage x pred** | **R2** | **RMSE** | **MAE** | **rage x pred** |
| **1** | E = 0.05  Dmax= 3  Tmax= 450 | 0.503 (0.087) | 5.300 (0.599) | 4.283 (0.555) | 0.719 [0.665, 0.766] | 0.621 (0.012) | 4.693 (0.071) | 3.759 (0.070) | 0.788 [0.783, 0.794] |
| **2** | E = 0.05  Dmax= 3  Tmax= 350 | 0.551 (0.081) | 5.107 (0.517) | 4.172 (0.474) | 0.750 [0.716, 0.780] | 0.613 (0.011) | 4.743 (0.071) | 3.803 (0.073) | 0.783 [0.778, 0.789] |
| **3** | E = 0.05  Dmax= 3  Tmax= 800 | 0.549 (0.078) | 5.129 (0.401) | 4.171 (0.393) | 0.751 [0.724, 0.776] | 0.635 (0.012) | 4.605 (0.067) | 3.683 (0.065) | 0.797 [0.792, 0.802] |
| **4** | E = 0.05  Dmax= 4  Tmax= 200 | 0.561 (0.062) | 5.136 (0.325) | 4.090 (0.257) | 0.741 [0.716, 0.763] | 0.606 (0.011) | 4.784 (0.068) | 3.840 (0.071) | 0.780 [0.774, 0.785] |
| **5** | E = 0.05  Dmax= 3  Tmax= 300 | 0.586 (0.037) | 4.994 (0.217) | 4.043 (0.241) | 0.764 [0.744, 0.783] | 0.607 (0.012) | 4.779 (0.074) | 3.834 (0.074) | 0.780 [0.774, 0.785] |
| **6** | E = 0.05  Dmax= 4  Tmax= 800 | 0.576 (0.035) | 4.962 (0.210) | 3.953 (0.149) | 0.763 [0.745, 0.780] | 0.641 (0.012) | 4.569 (0.058) | 3.652 (0.057) | 0.801 [0.795, 0.806] |
| **7** | E = 0.05  Dmax= 3  Tmax= 900 | 0.592 (0.042) | 4.887 (0.204) | 3.930 (0.150) | 0.774 [0.757, 0.789] | 0.637 (0.012) | 4.591 (0.068) | 3.669 (0.064) | 0.799 [0.793, 0.804] |
| **8** | E = 0.01  Dmax= 4  Tmax= 950 | 0.598 (0.028) | 4.881 (0.212) | 3.920 (0.216) | 0.764 [0.749, 0.779] | 0.605 (0.011) | 4.790 (0.072) | 3.848 (0.072) | 0.779 [0.773, 0.785] |
| **9** | E = 0.05  Dmax= 4  Tmax= 950 | 0.591 (0.036) | 4.882 (0.279) | 3.917 (0.175) | 0.774 [0.760, 0.788] | 0.643 (0.011) | 4.554 (0.056) | 3.638 (0.055) | 0.802 [0.797, 0.807] |
| **10** | E = 0.05  Dmax= 3  Tmax= 750 | 0.598 (0.033) | 4.886 (0.259) | 3.899 (0.230) | 0.777 [0.764, 0.790] | 0.633 (0.012) | 4.614 (0.067) | 3.691 (0.064) | 0.796 [0.791, 0.802] |
| **20** | E = 0.05  Dmax= 5  Tmax= 600 | 0.619 (0.025) | 4.748 (0.080) | 3.754 (0.124) | 0.787 [0.778, 0.795] | 0.638 (0.011) | 4.587 (0.061) | 3.665 (0.060) | 0.799 [0.794, 0.804] |
| **30** | E = 0.05  Dmax= 4  Tmax= 800 | 0.633 (0.009) | 4.623 (0.066) | 3.693 (0.065) | 0.798 [0.791, 0.804] | 0.641 (0.012) | 4.569 (0.058) | 3.652 (0.057) | 0.801 [0.795, 0.806] |
| **40** | E = 0.05  Dmax= 5  Tmax= 400 | 0.641 (0.014) | 4.584 (0.088) | 3.628  (0.050) | 0.797 [0.791, 0.803] | 0.631 (0.011) | 4.628 (0.062) | 3.701 (0.061) | 0.795 [0.790, 0.800] |
| **50** | E = 0.05  Dmax= 5  Tmax= 850 | 0.637 (0.017) | 4.576 (0.068) | 3.630 (0.049) | 0.805 [0.799, 0.810] | 0.641 (0.012) | 4.566 (0.059) | 3.647 (0.059) | 0.801 [0.796, 0.806] |

R2 = variance explained, RMSE = root mean square error, MAE = mean absolute error,rage x pred = correlation of chronological and predicted age. Numbers in round brackets indicate standard deviations. Numbers in square brackets indicate confidence intervals. E = eta (learning rate), Dmax = maximum depth, Tmax = maximum number of trees. The best fitting model was selected via grid search focussed on RMSE.

**ST2: Differences between correlations of chronological and *corrected* predicted age across models with 95% confidence interval**

|  | **BRIA** | **DKI** | **DTI** | **SMT** | **mcSMT** | **WMTI** | **Mean** |
| --- | --- | --- | --- | --- | --- | --- | --- |
| **DKI** | 0.011  [0.009, 0.013] |  |  |  |  |  |  |
| **DTI** | 0.008  [0.007, 0.010] | 0.003  [0.002, 0.005] |  |  |  |  |  |
| **SMT** | 0.007  [0.006, 0.008] | 0.004  [0.003, 0.005] | 0.001 [-0.0004, 0.0024]4 |  |  |  |  |
| **mcSMT** | ≈0  [-0.001, 0.001]1 | 0.011  [0.009, 0.013] | 0.008  [0.006, 0.010] | 0.007  [0.006, 0.008] |  |  |  |
| **WMTI** | 0.010  [0.009, 0.012] | 0.001  [-0.0004, 0.0024]2 | 0.002  [0.001, 0.003] | 0.003  [0.002, 0.004] | 0.010  [0.009, 0.012] |  |  |
| **Mean** | 0.013  [0.011, 0.015] | 0.002  [0.0002, 0.038]3 | 0.005  [0.003, 0.007] | 0.006  [0.004, 0.007] | 0.013  [0.011, 0.015] | 0.003  [0.001, 0.005] |  |
| **FULL** | 0.015  [0.014, 0.017] | 0.004  [0.003, 0.005] | 0.007  [0.006, 0.008] | 0.008  [0.007, 0.009] | 0.015  [0.014, 0.017] | 0.005  [0.004, 0.006] | 0.002  [0.0004, 0.0036] |

Confidence Intervals are based on Zou 42⁠. Unmarked differences were significant at *p* < .001.

1 Hotelling’s 86⁠ *t*(32171) ≈ 0, *p* ≈ 1.

2 Hotelling’s (1940)⁠ *t*(32171) = 1.4232, *p* = .1547.

3 Hotelling’s (1940)⁠ *t*(32171) = 2.2174, *p* = .0266.

4 Hotelling’s (1940)⁠ *t*(32171) = 2.4176, *p* = .0156.

**ST3: Differences between correlations of *uncorrected* predicted and chronological age across diffusion approaches with 95% confidence interval**

|  | **BRIA** | **DKI** | **DTI** | **SMT** | **mcSMT** | **WMTI** | **Mean** |
| --- | --- | --- | --- | --- | --- | --- | --- |
| **DKI** | 0.012  [0.009, 0.015] |  |  |  |  |  |  |
| **DTI** | 0.014  [0.011, 0.017] | 0.0002  [-0.005, 0.001] |  |  |  |  |  |
| **SMT** | 0.013  [0.010, 0.016] | 0.025  [0.022, 0.029] | 0.027  [0.024, 0.030] |  |  |  |  |
| **mcSMT** | 0.021  [0.019, 0.023] | 0.033  [0.029, 0.037] | 0.035  [0.032, 0.038] | 0.008  [0.006, 0.011] |  |  |  |
| **WMTI** | 0.023  [0.020, 0.026] | 0.011  [0.008, 0.014] | 0.009  [0.007, 0.011] | 0.036  [0.033, 0.039] | 0.044  [0.041, 0.047] |  |  |
| **Mean** | 0.115  [0.110, 0.120] | 0.127  [0.121, 0.133] | 0.129  [0.124, 0.134] | 0.102  [0.097, 0.107] | 0.088  [0.083, 0.093] | 0.0940  [0.089, 0.099] |  |
| **Full** | 0.062  [0.059, 0.065] | 0.050  [0.048, 0.053] | 0.048  [0.046, 0.051] | 0.075  [0.072, 0.078] | 0.083  [0.080, 0.086] | 0.039  [0.037, 0.041] | 0.138  [0.133, 0.143] |

Confidence Intervals are based on Zou (2007)⁠. Unmarked differences were significant at *p* < .001.

* Hotelling’s (1940)⁠ *t*(34801) = 0.9648, *p* = 0.3347

**ST4: Fornix metrics' age sensitivity: comparing diffusion metric prediction models with and without age**

| **Model** | **Metric** | **Full1** | **Reduced2** | **χ2** | ***p*** | ***p*Holm** |
| --- | --- | --- | --- | --- | --- | --- |
| BRIA | vintra | 63395.90 | 56372.64 | 14046.51 | <.001 | <.001 |
| BRIA | vextra | 53488.40 | 47076.64 | 12823.52 | <.001 | <.001 |
| BRIA | vCSF | 32902.83 | 25747.10 | 14311.47 | <.001 | <.001 |
| BRIA | microRD | -304.68 | -7544.95 | 14480.54 | <.001 | <.001 |
| BRIA | microFA | 52365.03 | 45215.37 | 14299.32 | <.001 | <.001 |
| BRIA | microAx | 27419.04 | 23070.45 | 8697.18 | <.001 | <.001 |
| BRIA | microADC | 7489.46 | 297.02 | 14384.87 | <.001 | <.001 |
| BRIA | DRADextra | 88839.19 | 84573.36 | 8531.64 | <.001 | <.001 |
| BRIA | DAXintra | 62832.56 | 58452.18 | 8760.75 | <.001 | <.001 |
| BRIA | DAXextra | 69448.84 | 68536.57 | 1824.54 | <.001 | <.001 |
| DKI | RK | 17066.24 | 10008.43 | 14115.62 | <.001 | <.001 |
| DKI | AK | 73031.59 | 67042.47 | 11978.25 | <.001 | <.001 |
| DKI | MK | 42812.92 | 35761.79 | 14102.27 | <.001 | <.001 |
| DTI | FA | 51988.61 | 46349.96 | 11277.31 | <.001 | <.001 |
| DTI | MD | -6425.12 | -12850.70 | 12851.17 | <.001 | <.001 |
| DTI | RD | -9157.86 | -15294.22 | 12272.70 | <.001 | <.001 |
| DTI | AD | -3330.43 | -9581.75 | 12502.64 | <.001 | <.001 |
| SMT | smtFA | 28610.35 | 23053.56 | 11113.58 | <.001 | <.001 |
| SMT | smtLong | 343020.49 | 342243.25 | 1554.49 | <.001 | <.001 |
| SMT | smtMD | 253964.29 | 248676.32 | 10575.95 | <.001 | <.001 |
| SMT | smtTrans | 239584.11 | 234337.78 | 10492.66 | <.001 | <.001 |
| mcSMT | smtMCintra | 46725.61 | 39797.67 | 13855.88 | <.001 | <.001 |
| mcSMT | smtMCextraMD | 260580.57 | 253725.97 | 13709.21 | <.001 | <.001 |
| mcSMT | smtMCextratrans | 249976.89 | 242973.81 | 14006.16 | <.001 | <.001 |
| mcSMT | smtMCd | 287624.52 | 284705.87 | 5837.30 | <.001 | <.001 |
| WMTI | AWF | 73001.96 | 65850.35 | 14303.22 | <.001 | <.001 |
| WMTI | axEAD | -28343.53 | -29855.90 | 3024.74 | <.001 | <.001 |
| WMTI | radEAD | -10582.93 | -16576.91 | 11987.96 | <.001 | <.001 |

1 Full = full model log likelihood

2 Reduced = reduced model log likelihood

**ST5: Model summaries for all 28 Fornix models**

| **Effect** | **β** | **Std. Error** | ***t*-value** | ***p*** | **Metric** | **β** | **Std. Error** | ***t*-value** | ***p*** | **Metric** |
| --- | --- | --- | --- | --- | --- | --- | --- | --- | --- | --- |
| age | 0.002 | 0.001 | 3.521 | 0 | vintra | 0.002 | 0.001 | 3.521 | 0 | vextra |
| age2 | 0 | 0 | -10.532 | 0 | vintra | 0 | 0 | -10.532 | 0 | vextra |
| sex | 0.009 | 0.018 | 0.481 | 0.63 | vintra | 0.009 | 0.018 | 0.481 | 0.63 | vextra |
| siteCheadle | 0.01 | 0.012 | 0.825 | 0.41 | vintra | 0.01 | 0.012 | 0.825 | 0.41 | vextra |
| siteNewcastle | 0.004 | 0.012 | 0.327 | 0.744 | vintra | 0.004 | 0.012 | 0.327 | 0.744 | vextra |
| siteReading | 0.009 | 0.012 | 0.748 | 0.454 | vintra | 0.009 | 0.012 | 0.748 | 0.454 | vextra |
| age:sex | 0 | 0 | -5.532 | 0 | vintra | 0 | 0 | -5.532 | 0 | vextra |
| sex:siteCheadle | -0.003 | 0.017 | -0.183 | 0.854 | vintra | -0.003 | 0.017 | -0.183 | 0.854 | vextra |
| sex:siteNewcastle | -0.003 | 0.017 | -0.168 | 0.867 | vintra | -0.003 | 0.017 | -0.168 | 0.867 | vextra |
| sex:siteReading | -0.005 | 0.017 | -0.283 | 0.777 | vintra | -0.005 | 0.017 | -0.283 | 0.777 | vextra |
| age | 0.002 | 0.001 | 3.521 | 0 | vCSF | 0.002 | 0.001 | 3.521 | 0 | microRD |
| age2 | 0 | 0 | -10.532 | 0 | vCSF | 0 | 0 | -10.532 | 0 | microRD |
| sex | 0.009 | 0.018 | 0.481 | 0.63 | vCSF | 0.009 | 0.018 | 0.481 | 0.63 | microRD |
| siteCheadle | 0.01 | 0.012 | 0.825 | 0.41 | vCSF | 0.01 | 0.012 | 0.825 | 0.41 | microRD |
| siteNewcastle | 0.004 | 0.012 | 0.327 | 0.744 | vCSF | 0.004 | 0.012 | 0.327 | 0.744 | microRD |
| siteReading | 0.009 | 0.012 | 0.748 | 0.454 | vCSF | 0.009 | 0.012 | 0.748 | 0.454 | microRD |
| age:sex | 0 | 0 | -5.532 | 0 | vCSF | 0 | 0 | -5.532 | 0 | microRD |
| sex:siteCheadle | -0.003 | 0.017 | -0.183 | 0.854 | vCSF | -0.003 | 0.017 | -0.183 | 0.854 | microRD |
| sex:siteNewcastle | -0.003 | 0.017 | -0.168 | 0.867 | vCSF | -0.003 | 0.017 | -0.168 | 0.867 | microRD |
| sex:siteReading | -0.005 | 0.017 | -0.283 | 0.777 | vCSF | -0.005 | 0.017 | -0.283 | 0.777 | microRD |
| age | 0.002 | 0.001 | 3.521 | 0 | microFA | 0.002 | 0.001 | 3.521 | 0 | microAx |
| age2 | 0 | 0 | -10.532 | 0 | microFA | 0 | 0 | -10.532 | 0 | microAx |
| sex | 0.009 | 0.018 | 0.481 | 0.63 | microFA | 0.009 | 0.018 | 0.481 | 0.63 | microAx |
| siteCheadle | 0.01 | 0.012 | 0.825 | 0.41 | microFA | 0.01 | 0.012 | 0.825 | 0.41 | microAx |
| siteNewcastle | 0.004 | 0.012 | 0.327 | 0.744 | microFA | 0.004 | 0.012 | 0.327 | 0.744 | microAx |
| siteReading | 0.009 | 0.012 | 0.748 | 0.454 | microFA | 0.009 | 0.012 | 0.748 | 0.454 | microAx |
| age:sex | 0 | 0 | -5.532 | 0 | microFA | 0 | 0 | -5.532 | 0 | microAx |
| sex:siteCheadle | -0.003 | 0.017 | -0.183 | 0.854 | microFA | -0.003 | 0.017 | -0.183 | 0.854 | microAx |
| sex:siteNewcastle | -0.003 | 0.017 | -0.168 | 0.867 | microFA | -0.003 | 0.017 | -0.168 | 0.867 | microAx |
| sex:siteReading | -0.005 | 0.017 | -0.283 | 0.777 | microFA | -0.005 | 0.017 | -0.283 | 0.777 | microAx |
| age | 0.002 | 0.001 | 3.521 | 0 | microADC | 0.002 | 0.001 | 3.521 | 0 | DRADextra |
| age2 | 0 | 0 | -10.532 | 0 | microADC | 0 | 0 | -10.532 | 0 | DRADextra |
| sex | 0.009 | 0.018 | 0.481 | 0.63 | microADC | 0.009 | 0.018 | 0.481 | 0.63 | DRADextra |
| siteCheadle | 0.01 | 0.012 | 0.825 | 0.41 | microADC | 0.01 | 0.012 | 0.825 | 0.41 | DRADextra |
| siteNewcastle | 0.004 | 0.012 | 0.327 | 0.744 | microADC | 0.004 | 0.012 | 0.327 | 0.744 | DRADextra |
| siteReading | 0.009 | 0.012 | 0.748 | 0.454 | microADC | 0.009 | 0.012 | 0.748 | 0.454 | DRADextra |
| age:sex | 0 | 0 | -5.532 | 0 | microADC | 0 | 0 | -5.532 | 0 | DRADextra |
| sex:siteCheadle | -0.003 | 0.017 | -0.183 | 0.854 | microADC | -0.003 | 0.017 | -0.183 | 0.854 | DRADextra |
| sex:siteNewcastle | -0.003 | 0.017 | -0.168 | 0.867 | microADC | -0.003 | 0.017 | -0.168 | 0.867 | DRADextra |
| sex:siteReading | -0.005 | 0.017 | -0.283 | 0.777 | microADC | -0.005 | 0.017 | -0.283 | 0.777 | DRADextra |
| age | 0.002 | 0.001 | 3.521 | 0 | DAXintra | 0.002 | 0.001 | 3.521 | 0 | DAXextra |
| age2 | 0 | 0 | -10.532 | 0 | DAXintra | 0 | 0 | -10.532 | 0 | DAXextra |
| sex | 0.009 | 0.018 | 0.481 | 0.63 | DAXintra | 0.009 | 0.018 | 0.481 | 0.63 | DAXextra |
| siteCheadle | 0.01 | 0.012 | 0.825 | 0.41 | DAXintra | 0.01 | 0.012 | 0.825 | 0.41 | DAXextra |
| siteNewcastle | 0.004 | 0.012 | 0.327 | 0.744 | DAXintra | 0.004 | 0.012 | 0.327 | 0.744 | DAXextra |
| siteReading | 0.009 | 0.012 | 0.748 | 0.454 | DAXintra | 0.009 | 0.012 | 0.748 | 0.454 | DAXextra |
| age:sex | 0 | 0 | -5.532 | 0 | DAXintra | 0 | 0 | -5.532 | 0 | DAXextra |
| sex:siteCheadle | -0.003 | 0.017 | -0.183 | 0.854 | DAXintra | -0.003 | 0.017 | -0.183 | 0.854 | DAXextra |
| sex:siteNewcastle | -0.003 | 0.017 | -0.168 | 0.867 | DAXintra | -0.003 | 0.017 | -0.168 | 0.867 | DAXextra |
| sex:siteReading | -0.005 | 0.017 | -0.283 | 0.777 | DAXintra | -0.005 | 0.017 | -0.283 | 0.777 | DAXextra |
| age | 0.002 | 0.001 | 3.521 | 0 | RK | 0.002 | 0.001 | 3.521 | 0 | AK |
| age2 | 0 | 0 | -10.532 | 0 | RK | 0 | 0 | -10.532 | 0 | AK |
| sex | 0.009 | 0.018 | 0.481 | 0.63 | RK | 0.009 | 0.018 | 0.481 | 0.63 | AK |
| siteCheadle | 0.01 | 0.012 | 0.825 | 0.41 | RK | 0.01 | 0.012 | 0.825 | 0.41 | AK |
| siteNewcastle | 0.004 | 0.012 | 0.327 | 0.744 | RK | 0.004 | 0.012 | 0.327 | 0.744 | AK |
| siteReading | 0.009 | 0.012 | 0.748 | 0.454 | RK | 0.009 | 0.012 | 0.748 | 0.454 | AK |
| age:sex | 0 | 0 | -5.532 | 0 | RK | 0 | 0 | -5.532 | 0 | AK |
| sex:siteCheadle | -0.003 | 0.017 | -0.183 | 0.854 | RK | -0.003 | 0.017 | -0.183 | 0.854 | AK |
| sex:siteNewcastle | -0.003 | 0.017 | -0.168 | 0.867 | RK | -0.003 | 0.017 | -0.168 | 0.867 | AK |
| sex:siteReading | -0.005 | 0.017 | -0.283 | 0.777 | RK | -0.005 | 0.017 | -0.283 | 0.777 | AK |
| age | 0.002 | 0.001 | 3.521 | 0 | MK | 0.002 | 0.001 | 3.521 | 0 | FA |
| age2 | 0 | 0 | -10.532 | 0 | MK | 0 | 0 | -10.532 | 0 | FA |
| sex | 0.009 | 0.018 | 0.481 | 0.63 | MK | 0.009 | 0.018 | 0.481 | 0.63 | FA |
| siteCheadle | 0.01 | 0.012 | 0.825 | 0.41 | MK | 0.01 | 0.012 | 0.825 | 0.41 | FA |
| siteNewcastle | 0.004 | 0.012 | 0.327 | 0.744 | MK | 0.004 | 0.012 | 0.327 | 0.744 | FA |
| siteReading | 0.009 | 0.012 | 0.748 | 0.454 | MK | 0.009 | 0.012 | 0.748 | 0.454 | FA |
| age:sex | 0 | 0 | -5.532 | 0 | MK | 0 | 0 | -5.532 | 0 | FA |
| sex:siteCheadle | -0.003 | 0.017 | -0.183 | 0.854 | MK | -0.003 | 0.017 | -0.183 | 0.854 | FA |
| sex:siteNewcastle | -0.003 | 0.017 | -0.168 | 0.867 | MK | -0.003 | 0.017 | -0.168 | 0.867 | FA |
| sex:siteReading | -0.005 | 0.017 | -0.283 | 0.777 | MK | -0.005 | 0.017 | -0.283 | 0.777 | FA |
| age | 0.002 | 0.001 | 3.521 | 0 | MD | 0.002 | 0.001 | 3.521 | 0 | RD |
| age2 | 0 | 0 | -10.532 | 0 | MD | 0 | 0 | -10.532 | 0 | RD |
| sex | 0.009 | 0.018 | 0.481 | 0.63 | MD | 0.009 | 0.018 | 0.481 | 0.63 | RD |
| siteCheadle | 0.01 | 0.012 | 0.825 | 0.41 | MD | 0.01 | 0.012 | 0.825 | 0.41 | RD |
| siteNewcastle | 0.004 | 0.012 | 0.327 | 0.744 | MD | 0.004 | 0.012 | 0.327 | 0.744 | RD |
| siteReading | 0.009 | 0.012 | 0.748 | 0.454 | MD | 0.009 | 0.012 | 0.748 | 0.454 | RD |
| age:sex | 0 | 0 | -5.532 | 0 | MD | 0 | 0 | -5.532 | 0 | RD |
| sex:siteCheadle | -0.003 | 0.017 | -0.183 | 0.854 | MD | -0.003 | 0.017 | -0.183 | 0.854 | RD |
| sex:siteNewcastle | -0.003 | 0.017 | -0.168 | 0.867 | MD | -0.003 | 0.017 | -0.168 | 0.867 | RD |
| sex:siteReading | -0.005 | 0.017 | -0.283 | 0.777 | MD | -0.005 | 0.017 | -0.283 | 0.777 | RD |
| age | 0.002 | 0.001 | 3.521 | 0 | AD | 0.002 | 0.001 | 3.521 | 0 | smtFA |
| age2 | 0 | 0 | -10.532 | 0 | AD | 0 | 0 | -10.532 | 0 | smtFA |
| sex | 0.009 | 0.018 | 0.481 | 0.63 | AD | 0.009 | 0.018 | 0.481 | 0.63 | smtFA |
| siteCheadle | 0.01 | 0.012 | 0.825 | 0.41 | AD | 0.01 | 0.012 | 0.825 | 0.41 | smtFA |
| siteNewcastle | 0.004 | 0.012 | 0.327 | 0.744 | AD | 0.004 | 0.012 | 0.327 | 0.744 | smtFA |
| siteReading | 0.009 | 0.012 | 0.748 | 0.454 | AD | 0.009 | 0.012 | 0.748 | 0.454 | smtFA |
| age:sex | 0 | 0 | -5.532 | 0 | AD | 0 | 0 | -5.532 | 0 | smtFA |
| sex:siteCheadle | -0.003 | 0.017 | -0.183 | 0.854 | AD | -0.003 | 0.017 | -0.183 | 0.854 | smtFA |
| sex:siteNewcastle | -0.003 | 0.017 | -0.168 | 0.867 | AD | -0.003 | 0.017 | -0.168 | 0.867 | smtFA |
| sex:siteReading | -0.005 | 0.017 | -0.283 | 0.777 | AD | -0.005 | 0.017 | -0.283 | 0.777 | smtFA |
| age | 0.002 | 0.001 | 3.521 | 0 | smtLong | 0.002 | 0.001 | 3.521 | 0 | smtMD |
| age2 | 0 | 0 | -10.532 | 0 | smtLong | 0 | 0 | -10.532 | 0 | smtMD |
| sex | 0.009 | 0.018 | 0.481 | 0.63 | smtLong | 0.009 | 0.018 | 0.481 | 0.63 | smtMD |
| siteCheadle | 0.01 | 0.012 | 0.825 | 0.41 | smtLong | 0.01 | 0.012 | 0.825 | 0.41 | smtMD |
| siteNewcastle | 0.004 | 0.012 | 0.327 | 0.744 | smtLong | 0.004 | 0.012 | 0.327 | 0.744 | smtMD |
| siteReading | 0.009 | 0.012 | 0.748 | 0.454 | smtLong | 0.009 | 0.012 | 0.748 | 0.454 | smtMD |
| age:sex | 0 | 0 | -5.532 | 0 | smtLong | 0 | 0 | -5.532 | 0 | smtMD |
| sex:siteCheadle | -0.003 | 0.017 | -0.183 | 0.854 | smtLong | -0.003 | 0.017 | -0.183 | 0.854 | smtMD |
| sex:siteNewcastle | -0.003 | 0.017 | -0.168 | 0.867 | smtLong | -0.003 | 0.017 | -0.168 | 0.867 | smtMD |
| sex:siteReading | -0.005 | 0.017 | -0.283 | 0.777 | smtLong | -0.005 | 0.017 | -0.283 | 0.777 | smtMD |
| age | 0.002 | 0.001 | 3.521 | 0 | smtTrans | 0.002 | 0.001 | 3.521 | 0 | smtMCintra |
| age2 | 0 | 0 | -10.532 | 0 | smtTrans | 0 | 0 | -10.532 | 0 | smtMCintra |
| sex | 0.009 | 0.018 | 0.481 | 0.63 | smtTrans | 0.009 | 0.018 | 0.481 | 0.63 | smtMCintra |
| siteCheadle | 0.01 | 0.012 | 0.825 | 0.41 | smtTrans | 0.01 | 0.012 | 0.825 | 0.41 | smtMCintra |
| siteNewcastle | 0.004 | 0.012 | 0.327 | 0.744 | smtTrans | 0.004 | 0.012 | 0.327 | 0.744 | smtMCintra |
| siteReading | 0.009 | 0.012 | 0.748 | 0.454 | smtTrans | 0.009 | 0.012 | 0.748 | 0.454 | smtMCintra |
| age:sex | 0 | 0 | -5.532 | 0 | smtTrans | 0 | 0 | -5.532 | 0 | smtMCintra |
| sex:siteCheadle | -0.003 | 0.017 | -0.183 | 0.854 | smtTrans | -0.003 | 0.017 | -0.183 | 0.854 | smtMCintra |
| sex:siteNewcastle | -0.003 | 0.017 | -0.168 | 0.867 | smtTrans | -0.003 | 0.017 | -0.168 | 0.867 | smtMCintra |
| sex:siteReading | -0.005 | 0.017 | -0.283 | 0.777 | smtTrans | -0.005 | 0.017 | -0.283 | 0.777 | smtMCintra |
| age | 0.002 | 0.001 | 3.521 | 0 | smtMCextraMD | 0.002 | 0.001 | 3.521 | 0 | smtMCextratrans |
| age2 | 0 | 0 | -10.532 | 0 | smtMCextraMD | 0 | 0 | -10.532 | 0 | smtMCextratrans |
| sex | 0.009 | 0.018 | 0.481 | 0.63 | smtMCextraMD | 0.009 | 0.018 | 0.481 | 0.63 | smtMCextratrans |
| siteCheadle | 0.01 | 0.012 | 0.825 | 0.41 | smtMCextraMD | 0.01 | 0.012 | 0.825 | 0.41 | smtMCextratrans |
| siteNewcastle | 0.004 | 0.012 | 0.327 | 0.744 | smtMCextraMD | 0.004 | 0.012 | 0.327 | 0.744 | smtMCextratrans |
| siteReading | 0.009 | 0.012 | 0.748 | 0.454 | smtMCextraMD | 0.009 | 0.012 | 0.748 | 0.454 | smtMCextratrans |
| age:sex | 0 | 0 | -5.532 | 0 | smtMCextraMD | 0 | 0 | -5.532 | 0 | smtMCextratrans |
| sex:siteCheadle | -0.003 | 0.017 | -0.183 | 0.854 | smtMCextraMD | -0.003 | 0.017 | -0.183 | 0.854 | smtMCextratrans |
| sex:siteNewcastle | -0.003 | 0.017 | -0.168 | 0.867 | smtMCextraMD | -0.003 | 0.017 | -0.168 | 0.867 | smtMCextratrans |
| sex:siteReading | -0.005 | 0.017 | -0.283 | 0.777 | smtMCextraMD | -0.005 | 0.017 | -0.283 | 0.777 | smtMCextratrans |
| age | 0.002 | 0.001 | 3.521 | 0 | smtMCd | 0.002 | 0.001 | 3.521 | 0 | AWF |
| age2 | 0 | 0 | -10.532 | 0 | smtMCd | 0 | 0 | -10.532 | 0 | AWF |
| sex | 0.009 | 0.018 | 0.481 | 0.63 | smtMCd | 0.009 | 0.018 | 0.481 | 0.63 | AWF |
| siteCheadle | 0.01 | 0.012 | 0.825 | 0.41 | smtMCd | 0.01 | 0.012 | 0.825 | 0.41 | AWF |
| siteNewcastle | 0.004 | 0.012 | 0.327 | 0.744 | smtMCd | 0.004 | 0.012 | 0.327 | 0.744 | AWF |
| siteReading | 0.009 | 0.012 | 0.748 | 0.454 | smtMCd | 0.009 | 0.012 | 0.748 | 0.454 | AWF |
| age:sex | 0 | 0 | -5.532 | 0 | smtMCd | 0 | 0 | -5.532 | 0 | AWF |
| sex:siteCheadle | -0.003 | 0.017 | -0.183 | 0.854 | smtMCd | -0.003 | 0.017 | -0.183 | 0.854 | AWF |
| sex:siteNewcastle | -0.003 | 0.017 | -0.168 | 0.867 | smtMCd | -0.003 | 0.017 | -0.168 | 0.867 | AWF |
| sex:siteReading | -0.005 | 0.017 | -0.283 | 0.777 | smtMCd | -0.005 | 0.017 | -0.283 | 0.777 | AWF |
| age | 0.002 | 0.001 | 3.521 | 0 | axEAD | 0.002 | 0.001 | 3.521 | 0 | radEAD |
| age2 | 0 | 0 | -10.532 | 0 | axEAD | 0 | 0 | -10.532 | 0 | radEAD |
| sex | 0.009 | 0.018 | 0.481 | 0.63 | axEAD | 0.009 | 0.018 | 0.481 | 0.63 | radEAD |
| siteCheadle | 0.01 | 0.012 | 0.825 | 0.41 | axEAD | 0.01 | 0.012 | 0.825 | 0.41 | radEAD |
| siteNewcastle | 0.004 | 0.012 | 0.327 | 0.744 | axEAD | 0.004 | 0.012 | 0.327 | 0.744 | radEAD |
| siteReading | 0.009 | 0.012 | 0.748 | 0.454 | axEAD | 0.009 | 0.012 | 0.748 | 0.454 | radEAD |
| age:sex | 0 | 0 | -5.532 | 0 | axEAD | 0 | 0 | -5.532 | 0 | radEAD |
| sex:siteCheadle | -0.003 | 0.017 | -0.183 | 0.854 | axEAD | -0.003 | 0.017 | -0.183 | 0.854 | radEAD |
| sex:siteNewcastle | -0.003 | 0.017 | -0.168 | 0.867 | axEAD | -0.003 | 0.017 | -0.168 | 0.867 | radEAD |
| sex:siteReading | -0.005 | 0.017 | -0.283 | 0.777 | axEAD | -0.005 | 0.017 | -0.283 | 0.777 | radEAD |

**ST6: Brain age prediction model performance *for* *data including QC outliers***

| **Name** | **MRI features** | **R2** | **RMSE** | **MAE** | **Prediction-Age Correlation*** | **Corrected Prediction-Age Correlation*** |
| --- | --- | --- | --- | --- | --- | --- |
| BRIA | 700 | 0.538  (0.009) | 5.103  (0.044) | 4.096  (0.038) | 0.734  [0.729, 0.739] | 0.902  [0.900, 0.904] |
| DKI | 210 | 0.561  (0.009) | 5.078  (0.053) | 4.073  (0.047) | 0.750  [0.745, 0.754] | 0.876  [0.874, 0.879] |
| DTI | 280 | 0.565  (0.009) | 5.052  (0.039) | 4.041  (0.038) | 0.752  [0.748, 0.757] | 0.874  [0.872, 0.877] |
| SMT | 280 | 0.522  (0.009) | 5.297  (0.035) | 4.254  (0.031) | 0.723  [0.718, 0.728] | 0.870  [0.868, 0.873] |
| mcSMT | 280 | 0.508  (0.008) | 5.263  (0.040) | 4.227  (0.034) | 0.714  [0.708, 0.719] | 0.901  [0.899, 0.903] |
| WMTI | 210 | 0.574  (0.009) | 4.999  (0.036) | 4.003  (0.034) | 0.758  [0.754, 0.763] | 0.875  [0.873, 0.877] |
| Mean scores multimodal | 28 | 0.400  (0.068) | 5.945  (0.082) | 4.820  (0.068) | 0.633  [0.627, 0.639] | 0.875  [0.873, 0.878] |
| Full model multimodal | 1932 | 0.648  (0.009) | 4.557  (0.077) | 3.637  (0.066) | 0.805  [0.801, 0.808] | 0.877  [0.875, 0.880] |

Model selection was based on a grid search with stopping rule when model performance did not not improve after 20 rounds. Model selection of all models was based on multimodal model training on 10% of the data, indicating best fit for learning rate = 0.05, maximum depth = 4, maximum number of trees = 750 as indicated in Fig.2 and ST1.

R2:variance explained, RMSE: root mean squared error, MAE: mean absolute error

*Note*: R2, RMSE, MAE are displayed in the format Mean (Standard Deviation), Pearson’s correlations are displayed in the format Correlation Score 95% Confidence Interval [Lower Bound, Upper Bound].

*All correlation were significant at *p* < .001.

Outliers were defined by the YTTRIUM method38⁠ including outlier removal based on density-based spatial clusterisation (k-means). The total data used here was Nfull+outliers = 38,687, including the full data Nfull = 35,749 used for all analyses and Noutliers = 2,938 datasets defined as outliers. This dataset does not include participants who withdrew their consent or participants with an ICD-10 diagnosis categories G or F or stroke, category I.

**ST7: Top five diffusion metrics ranked by gain in age prediction accuracy *for data including QC outliers***

| **BRIA** | **DKI** | **DTI** | **SMT** | **mcSMT** | **WMTI** | **Full** |
| --- | --- | --- | --- | --- | --- | --- |
| Micro RD ATRR (67436) | MK Fornix (99885) | MD Fornix (79767) | MD Fornix (72129) | Extratrans Fornix  (55429) | AWF Fornix  (69033) | AWF fornix  (278977) |
| Micro FA Fornix (63804) | RK Fornix (28196) | RD left anterior corona radiata (51592) | MD FMIN (48161) | Intra Fornix (46212) | RadEAD anterior right corona radiata (34381) | Micro RD fornix (71175) |
| Micro RD right external capsule (27069) | AK anterior right limb of internal capsule  (22725) | RD FMIN (25201) | MD right anterior corona radiata  (44331) | Extratrans right external capsule (16611) | RadEAD IFOFR (22512) | Micro FA fornix(35049) |
| Micro RD Fornix right striaterminalis (17090) | AK Fornix (14401) | RD Fornix right stria terminalis (22951) | FA Fornix (19697) | ExtraMD Fornix (10267) | RadEAD FMIN (20286) | MD right tapetum  (34008) |
| Micro FA FMIN (14335) | AK superior frontooccipital left fasciculus (7978) | MD anterior limb of internal left capsule (15589) | Long left tapetum  (14596) | ExtraMD anterior left limb of internal capsule  (7830) | RadEAD ATRL (16666) | RadEAD anterior right corona radiata (27907) |

Table values can be read as feature name (gain). Mean refers to the multimodal model containing only mean scores and full to the full model containing all features. Cells including Fornix are marked in green.

laLC = left anterior limb of internal capsule; raLC = right anterior limb of internal capsule; lST = lSTria terminalis; rST = rSTria terminalis; lsfoF = left superior frontal occipital fasciculus; laCR = left anterior corona radiata; raCR = right anterior corona radiata; rEC = right external capsule

Outliers were defined by the YTTRIUM method38⁠ including outlier removal based on density-based spatial clusterisation (k-means). The total data used here was Nfull+outliers = 38,687, including the full data Nfull = 35,749 used for all analyses and Noutliers = 2,938 datasets defined as outliers. This dataset does not include participants who withdrew their consent or participants with an ICD-10 diagnosis categories G or F or stroke, category I.

**ST8: Brain age predictions from different train-test splits *for data including QC outliers***

| **% of Data** | **Best Fitting Model** | **Train Results** | | | | **Test Results on 50% of Data** | | | |
| --- | --- | --- | --- | --- | --- | --- | --- | --- | --- |
|  |  | **R2** | **RMSE** | **MAE** | **rage x pred** | **R2** | **RMSE** | **MAE** | **rage x pred** |
| **1** | E = 0.05  Dmax= 4  Tmax= 250 | 0.503 (0.113) | 5.313 (0.448) | 4.287 (0.233) | 0.716 [0.664, 0.761] | 0.619 (0.008) | 4.743 (0.029) | 3.799 (0.036) | 0.788 [0.782, 0.793] |
| **2** | E = 0.05  Dmax= 4  Tmax= 950 | 0.543 (0.094) | 5.162 (0.365) | 4.101 (0.250) | 0.744 [0.711, 0.774] | 0.648 (0.008) | 4.558 (0.025) | 3.637 (0.025) | 0.805 [0.800, 0.810] |
| **3** | E = 0.05  Dmax= 4  Tmax= 900 | 0.543 (0.052) | 5.226 (0.334) | 4.149 (0.297) | 0.741 [0.714, 0.766] | 0.648 (0.008) | 4.561 (0.024) | 3.639 (0.026) | 0.805 [0.800, 0.810] |
| **4** | E = 0.05  Dmax= 3  Tmax= 550 | 0.572 (0.033) | 5.110 (0.266) | 4.043 (0.184) | 0.758 [0.736, 0.779] | 0.619 (0.008) | 4.744 (0.036) | 3.803 (0.041) | 0.788 [0.782, 0.793] |
| **5** | E = 0.05  Dmax= 3  Tmax= 350 | 0.575 (0.051) | 5.038 (0.312) | 4.015 (0.233) | 0.760 [0.740, 0.778] | 0.619 (0.008) | 4.744 (0.036) | 3.803 (0.041) | 0.788 [0.782, 0.793] |
| **6** | E = 0.05  Dmax= 4  Tmax= 850 | 0.574 (0.047) | 5.144 (0.239) | 4.048 (0.195) | 0.758 [0.740, 0.774] | 0.647 (0.008) | 4.566 (0.024) | 3.643 (0.026) | 0.805 [0.800, 0.810] |
| **7** | E = 0.05  Dmax= 3  Tmax= 900 | 0.589 (0.032) | 4.998 (0.147) | 4.004 (0.186) | 0.770 [0.754, 0.785] | 0.644 (0.008) | 4.585 (0.039) | 3.663 (0.040) | 0.803 [0.798, 0.808] |
| **8** | E = 0.05  Dmax= 3  Tmax= 550 | 0.595 (0.017) | 4.944 (0.174) | 3.953 (0.138) | 0.775 [0.761, 0.789] | 0.644 (0.008) | 4.585 (0.039) | 3.663 (0.040) | 0.803 [0.798, 0.808] |
| **9** | E = 0.05  Dmax= 3  Tmax= 350 | 0.602 (0.025) | 4.915 (0.195) | 3.921 (0.169) | 0.773 [0.759, 0.786] | 0.619 (0.008) | 4.744 (0.036) | 3.803 (0.041) | 0.788 [0.782, 0.793] |
| **10** | E = 0.05  Dmax= 3  Tmax= 550 | 0.608 (0.033) | 4.869 (0.188) | 3.882 (0.114) | 0.780 [0.768, 0.792] | 0.628 (0.008) | 4.690 (0.037) | 3.756 (0.041) | 0.793 [0.788, 0.798] |
| **20** | E = 0.05  Dmax= 4  Tmax= 450 | 0.624 (0.013) | 4.706 (0.097) | 3.779 (0.092) | 0.791 [0.782, 0.799] | 0.640 (0.008) | 4.608 (0.024) | 3.679 (0.028) | 0.801 [0.796, 0.806] |
| **30** | E = 0.05  Dmax= 4  Tmax= 900 | 0.641 (0.017) | 4.611 (0.066) | 3.683 (0.064) | 0.641 [0.798, 0.811] | 0.636 (0.008) | 4.634 (0.023) | 3.703 (0.029) | 0.798 [0.793, 0.803] |
| **40** | E = 0.05  Dmax= 5  Tmax= 950 | 0.643 (0.019) | 4.596 (0.063) | 3.668 (0.053) | 0.803 [0.797, 0.808] | 0.648 (0.009) | 4.557 (0.033) | 3.635 (0.037) | 0.806 [0.801, 0.810] |
| **50** | E = 0.05  Dmax= 4  Tmax= 850 | 0.643 (0.020) | 4.584 (0.122) | 3.637 (0.088) | 0.806 [0.801, 0.811] | 0.647 (0.008) | 4.566 (0.024) | 3.643 (0.026) | 0.805 [0.800, 0.810] |

Note: Numbers in round brackets indicate standard deviations. Numbers in square brackets indicate confidence intervals. E = eta, Dmax = maximum depth, Tmax = maximum number of trees. The best fitting model was determined by grid search.

Outliers were defined by the YTTRIUM method38⁠ including outlier removal based on density-based spatial clusterisation (k-means). The total data used here was Nfull+outliers = 38,687, including the full data Nfull = 35,749 used for all analyses and Noutliers = 2,938 datasets defined as outliers. This dataset does not include participants who withdrew their consent or participants with an ICD-10 diagnosis categories G or F or stroke, category I.

**ST9: Comparisons of linear and generalized additive models predicting fornix diffusion metrics**

| **Metric** | **LM AIC** | **GAM AIC** | **LM BIC** | **GAM BIC** | **LM R2adj** | **GAM R2adj** |
| --- | --- | --- | --- | --- | --- | --- |
| vintra | -126767.79 | -126755.60 | -126665.98 | -126662.27 | 0.36 | 0.36 |
| vextra | -106952.81 | -106929.99 | -106851.00 | -106836.66 | 0.38 | 0.37 |
| vCSF | -65781.67 | -65773.53 | -65679.86 | -65680.20 | 0.39 | 0.39 |
| microRD | 633.36 | 643.11 | 735.18 | 736.43 | 0.39 | 0.39 |
| microFA | -104706.06 | -104634.30 | -104604.25 | -104540.97 | 0.38 | 0.38 |
| microAx | -54814.08 | -54790.59 | -54712.27 | -54697.26 | 0.27 | 0.27 |
| microADC | -14954.91 | -14946.59 | -14853.10 | -14853.26 | 0.39 | 0.39 |
| DRADextra | -177654.37 | -177648.58 | -177552.56 | -177555.25 | 0.30 | 0.30 |
| DAXintra | -125641.12 | -125639.53 | -125539.31 | -125546.20 | 0.30 | 0.30 |
| DAXextra | -138873.67 | -138808.20 | -138771.86 | -138714.87 | 0.09 | 0.09 |
| RK | -34108.48 | -34109.41 | -34006.67 | -34016.08 | 0.37 | 0.37 |
| AK | -146039.18 | -146041.08 | -145937.37 | -145947.75 | 0.32 | 0.32 |
| MK | -85601.85 | -85603.57 | -85500.04 | -85510.24 | 0.36 | 0.36 |
| FA | -103953.22 | -103924.62 | -103851.41 | -103831.30 | 0.31 | 0.31 |
| MD | 12874.23 | 12878.93 | 12976.04 | 12972.25 | 0.35 | 0.35 |
| RD | 18339.73 | 18359.69 | 18441.54 | 18453.02 | 0.34 | 0.34 |
| AD | 6684.86 | 6689.75 | 6786.67 | 6783.08 | 0.35 | 0.35 |
| smtFA | -57196.69 | -57161.31 | -57094.88 | -57067.98 | 0.31 | 0.31 |
| smtLong | -686016.98 | -685869.48 | -685915.17 | -685776.15 | 0.06 | 0.05 |
| smtMD | -507904.58 | -507869.45 | -507802.77 | -507776.13 | 0.30 | 0.30 |
| smtTrans | -479144.23 | -479104.60 | -479042.42 | -479011.28 | 0.30 | 0.29 |
| smtMCintra | -93427.21 | -93427.11 | -93325.40 | -93333.78 | 0.36 | 0.36 |
| smtMCextraMD | -521137.14 | -521125.59 | -521035.33 | -521032.26 | 0.37 | 0.37 |
| smtMCextratrans | -499929.78 | -499929.66 | -499827.97 | -499836.34 | 0.37 | 0.37 |
| smtMCd | -575225.03 | -574996.85 | -575123.22 | -574903.53 | 0.22 | 0.21 |
| AWF | -145979.93 | -145981.86 | -145878.11 | -145888.53 | 0.37 | 0.37 |
| axEAD | 56711.05 | 56715.91 | 56812.86 | 56809.24 | 0.10 | 0.10 |
| radEAD | 21189.85 | 21203.80 | 21291.67 | 21297.12 | 0.33 | 0.33 |

LM = linear model, GAM = generalized additive model, AIC = Akaike information criterion, BIC = Bayesian information criterion. The numbers are derived from the six diffusion approaches’ 28 metrics following Equation 1 for linear models and all variables of the equation allowing splines for non-linear models.

**ST10: Overview of diffusion metrics by diffusion approach**

| **Diffusion Approach** | **Metrics** |
| --- | --- |
| Bayesian Rotationally Invariant Approach (BRIA) | intra-axonal axial diffusivity (DAX intra) |
|  | extra-axonal radial diffusivity (DRAD extra) |
|  | microscopic fractional anisotropy (micro FA) |
|  | extra-axonal axial diffusivity (DAX extra) |
|  | intra-axonal water fraction (V intra) |
|  | extra-axonal water fraction (V extra) |
|  | cerebrospinal fluid fraction (vCSF) |
|  | microscopical axial diffusivity (micro AX) |
|  | microscopic radial diffusivity (micro RD) |
|  | microscopical apparent diffusion coefficient (micro ADC) |
| Diffusion Kurtosis Imaging (DKI) | mean kurtosis (MK) |
|  | radial kurtosis (RK) |
|  | axial kurtosis (AK) |
| Diffusion Tensor Imaging (DTI) | fractional anisotropy (FA) |
|  | axial diffusivity (AD) |
|  | mean diffusivity (MD) |
|  | radial diffusivity (RD) |
| Spherical Mean Technique (SMT) | fractional anisotropy (SMT FA) |
|  | mean diffusivity (SMT md) |
|  | transverse diffusion coefficient (SMT trans) |
|  | longitudinal diffusion coefficient (SMT long) |
| Multi-compartment Spherical Mean Technique (mcSMT) | extra-neurite microscopic mean diffusivity (mcSMT extra md) |
|  | extra-neurite transverse microscopic diffusivity (mcSMT extra trans) |
|  | mc SMTdiffusion coefficient (SMT mcd) |
|  | intra-neurite volume fraction (mcSMT intra) |
| White Matter Tract Integrity (WMTI) | axonal water fraction (AWF) |
|  | radial extra-axonal diffusivity (radEAD) |
|  | axial extra-axonal diffusivity (axEAD) |

**ST11. Whole-brain metrics' age sensitivity: comparing diffusion metric prediction models with and without age**

| **Metric** | **Full1** | **Reduced2** | **χ2** | ***p*** | ***p*Holm** |
| --- | --- | --- | --- | --- | --- |
| vintra | 77831.60 | 75474.15 | 4714.90 | <.001 | <.001 |
| vextra | 80010.15 | 79172.34 | 1675.61 | <.001 | <.001 |
| vCSF | 104258.51 | 102045.76 | 4425.50 | <.001 | <.001 |
| microRD | 67462.88 | 62714.78 | 9496.19 | <.001 | <.001 |
| microFA | 91462.73 | 87450.56 | 8024.34 | <.001 | <.001 |
| microAx | 68450.93 | 67363.35 | 2175.17 | <.001 | <.001 |
| microADC | 72076.62 | 67934.52 | 8284.22 | <.001 | <.001 |
| DRADextra | 105039.68 | 102688.39 | 4702.58 | <.001 | <.001 |
| DAXintra | 66812.02 | 64826.61 | 3970.82 | <.001 | <.001 |
| DAXextra | 80174.07 | 77914.51 | 4519.12 | <.001 | <.001 |
| RK | 44127.87 | 41757.04 | 4741.66 | <.001 | <.001 |
| AK | 87172.98 | 85483.34 | 3379.29 | <.001 | <.001 |
| MK | 66245.64 | 64166.15 | 4158.99 | <.001 | <.001 |
| FA | 93186.73 | 88785.16 | 8803.13 | <.001 | <.001 |
| MD | 76490.81 | 72273.83 | 8433.95 | <.001 | <.001 |
| RD | 73140.15 | 68320.02 | 9640.26 | <.001 | <.001 |
| AD | 76516.84 | 74699.38 | 3634.93 | <.001 | <.001 |
| smtFA | 125878.97 | 124092.64 | 3572.65 | <.001 | <.001 |
| smtLong | 287398.36 | 285277.70 | 4241.31 | <.001 | <.001 |
| smtMD | 320273.27 | 317056.32 | 6433.91 | <.001 | <.001 |
| smtTrans | 342834.04 | 339946.36 | 5775.35 | <.001 | <.001 |
| smtMCintra | 74365.62 | 72478.15 | 3774.94 | <.001 | <.001 |
| smtMCextraMD | 314618.75 | 311214.26 | 6809.00 | <.001 | <.001 |
| smtMCextratrans | 303909.44 | 300508.10 | 6802.69 | <.001 | <.001 |
| smtMCd | 290508.70 | 290389.47 | 238.47 | <.001 | <.001 |
| AWF | 102689.44 | 100136.63 | 5105.61 | <.001 | <.001 |
| axEAD | -14423.53 | -14426.86 | 6.66 | 0.08 | 0.08 |
| radEAD | 10331.86 | 10122.73 | 418.26 | <.001 | <.001 |

1 Full = full model log likelihood

2 Reduced = reduced model log likelihood

**ST12. Comparisons of linear and generalized additive models predicting whole-brain diffusion metrics**

| **Metric** | **LM AIC** | **GAM AIC** | **LM BIC** | **GAM BIC** | **LM R2adj** | **GAM R2adj** |
| --- | --- | --- | --- | --- | --- | --- |
| vintra | -155639.20 | -155638.58 | -155537.39 | -155545.25 | 0.13 | 0.13 |
| vextra | -159996.29 | -159998.05 | -159894.48 | -159904.72 | 0.05 | 0.05 |
| vCSF | -208493.03 | -208487.27 | -208391.21 | -208393.94 | 0.13 | 0.13 |
| microRD | -134901.75 | -134894.29 | -134799.94 | -134800.96 | 0.25 | 0.25 |
| microFA | -182901.46 | -182896.42 | -182799.64 | -182803.09 | 0.21 | 0.21 |
| microAx | -136877.86 | -136869.60 | -136776.05 | -136776.28 | 0.08 | 0.08 |
| microADC | -144129.25 | -144118.74 | -144027.44 | -144025.42 | 0.23 | 0.23 |
| DRADextra | -210055.37 | -210046.77 | -209953.56 | -209953.44 | 0.13 | 0.13 |
| DAXintra | -133600.03 | -133591.04 | -133498.22 | -133497.71 | 0.11 | 0.11 |
| DAXextra | -160324.15 | -160312.75 | -160222.34 | -160219.42 | 0.13 | 0.13 |
| RK | -88231.74 | -88223.96 | -88129.93 | -88130.63 | 0.14 | 0.14 |
| AK | -174321.97 | -174323.26 | -174220.16 | -174229.93 | 0.09 | 0.09 |
| MK | -132467.28 | -132465.03 | -132365.47 | -132371.70 | 0.12 | 0.12 |
| FA | -186349.46 | -186338.20 | -186247.65 | -186244.88 | 0.23 | 0.23 |
| MD | -152957.62 | -152950.05 | -152855.81 | -152856.72 | 0.22 | 0.22 |
| RD | -146256.29 | -146248.25 | -146154.48 | -146154.92 | 0.24 | 0.24 |
| AD | -153009.69 | -153006.22 | -152907.88 | -152912.90 | 0.15 | 0.15 |
| smtFA | -251733.94 | -251735.36 | -251632.13 | -251642.03 | 0.10 | 0.10 |
| smtLong | -574772.72 | -574753.50 | -574670.90 | -574660.18 | 0.12 | 0.12 |
| smtMD | -640522.55 | -640508.56 | -640420.74 | -640415.23 | 0.18 | 0.18 |
| smtTrans | -685644.08 | -685645.94 | -685542.27 | -685552.61 | 0.16 | 0.16 |
| smtMCintra | -148707.24 | -148707.45 | -148605.43 | -148614.13 | 0.10 | 0.10 |
| smtMCextraMD | -629213.51 | -629198.88 | -629111.69 | -629105.56 | 0.18 | 0.18 |
| smtMCextratrans | -607794.88 | -607787.65 | -607693.07 | -607694.32 | 0.18 | 0.18 |
| smtMCd | -580993.40 | -580991.12 | -580891.59 | -580897.79 | 0.01 | 0.01 |
| AWF | -205354.88 | -205349.62 | -205253.07 | -205256.29 | 0.14 | 0.14 |
| axEAD | 28871.07 | 28869.86 | 28972.88 | 28963.19 | 0.00 | 0.00 |
| radEAD | -20639.72 | -20639.85 | -20537.91 | -20546.52 | 0.01 | 0.01 |

LM = linear model, GAM = generalized additive model, AIC = Akaike information criterion, BIC = Bayesian information criterion. The numbers are derived from the six diffusion approaches’ 28 metrics following Equation 1 for linear models and all variables of the equation allowing splines for non-linear models.

**ST13.** **Variance explained by principal components of white matter metrics**

|  | **Component 1** | **Component 2** | **Component 3** | **Component 4** | **Component 5** | **Component 6** | **Component 7** | **Component 8** | **Component 9** | **Component 10** |
| --- | --- | --- | --- | --- | --- | --- | --- | --- | --- | --- |
| **Full Multimodal** | 0.3500 | 0.1065 | 0.0583 | 0.0393 | 0.0328 | 0.0208 | 0.0193 | 0.0172 | 0.0144 | 0.0133 |
| **Mean Multimodal** | 0.6474 | 0.2085 | 0.0690 | 0.0354 | 0.0199 | 0.0069 | 0.0046 | 0.0029 | 0.0020 | 0.0010 |
| **BRIA** | 0.3759 | 0.0963 | 0.0706 | 0.0533 | 0.0377 | 0.0291 | 0.0238 | 0.0177 | 0.0140 | 0.0128 |
| **DKI** | 0.4358 | 0.0909 | 0.0479 | 0.0309 | 0.0294 | 0.0228 | 0.0174 | 0.0153 | 0.0137 | 0.0123 |
| **DTI** | 0.4072 | 0.0816 | 0.0532 | 0.0434 | 0.0353 | 0.0249 | 0.0206 | 0.0165 | 0.0145 | 0.0138 |
| **SMT** | 0.3393 | 0.1942 | 0.0603 | 0.0336 | 0.0255 | 0.0215 | 0.0207 | 0.0173 | 0.0166 | 0.0130 |
| **SMT mc** | 0.3404 | 0.1648 | 0.0585 | 0.0450 | 0.0329 | 0.0224 | 0.0203 | 0.0165 | 0.0155 | 0.0127 |
| **WMTI** | 0.2711 | 0.1277 | 0.0421 | 0.0379 | 0.0284 | 0.0279 | 0.0217 | 0.0195 | 0.0178 | 0.0168 |

Eight principal component analyses (PCA) were run: six PCA addressing the different diffusion approaches, one addressing the multimodal average scores (mean multimodal) and one the multimodal model, containing all data (full multimodal). The first four components from all PCA were deemed meaningful based on the proportion of variance explained in the WM data.

**ST14. Model performance and BAG beta values for multimodal and diffusion-approach specific principal component predictions from multimodal and diffusion approach-specific BAG and covariates**

| **Predicted Component** | **Approach** | **R2** | **Radj2** | ***T*** | ***p*** | ***b*BAG** |
| --- | --- | --- | --- | --- | --- | --- |
| 1 | Full Multimodal | 0.0141 | 0.0138 | 46.0160 | <0.0001 | -0.0151 |
| 1 | Mean Multimodal | 0.5054 | 0.5052 | 3651.2999 | <0.0001 | -0.6726 |
| 1 | BRIA | 0.3439 | 0.3437 | 1685.8645 | <0.0001 | -1.8093 |
| 1 | DKI | 0.2263 | 0.2261 | 940.7306 | <0.0001 | -0.8142 |
| 1 | DTI | 0.3575 | 0.3573 | 1789.7236 | <0.0001 | -1.0817 |
| 1 | SMT | 0.3462 | 0.3460 | 1841.6494 | <0.0001 | -1.0953 |
| 1 | SMT mc | 0.3152 | 0.3150 | 1480.6879 | <0.0001 | -1.0782 |
| 1 | WMTI | 0.3720 | 0.3718 | 1905.4699 | <0.0001 | -0.8474 |
| 2 | Full Multimodal | 0.0247 | 0.0244 | 81.5815 | <0.0001 | -0.0332 |
| 2 | Mean Multimodal | 0.0179 | 0.0177 | 65.2632 | <0.0001 | 0.0522 |
| 2 | BRIA | 0.0400 | 0.0397 | 134.1182 | <0.0001 | 0.1030 |
| 2 | DKI | 0.0191 | 0.0188 | 62.7254 | <0.0001 | 0.1090 |
| 2 | DTI | 0.1517 | 0.1514 | 575.2169 | <0.0001 | -0.0587 |
| 2 | SMT | 0.0022 | 0.0019 | 7.6338 | <0.0001 | -0.0159 |
| 2 | SMT mc | 0.0784 | 0.0781 | 273.5101 | <0.0001 | 0.2988 |
| 2 | WMTI | 0.0563 | 0.0560 | 191.8700 | <0.0001 | 0.0876 |
| 3 | Full Multimodal | 0.1148 | 0.1145 | 417.0057 | <0.0001 | -0.0086 |
| 3 | Mean Multimodal | 0.0003 | 0.0001 | 1.1795 | 0.2991 | -0.0023 |
| 3 | BRIA | 0.1842 | 0.1839 | 726.2248 | <0.0001 | 0.4627 |
| 3 | DKI | 0.2564 | 0.2562 | 1109.0911 | <0.0001 | 0.1705 |
| 3 | DTI | 0.2500 | 0.2498 | 1072.1366 | <0.0001 | -0.2096 |
| 3 | SMT | 0.2465 | 0.2463 | 1137.8183 | <0.0001 | 0.2908 |
| 3 | SMT mc | 0.1844 | 0.1841 | 726.9496 | <0.0001 | -0.1839 |
| 3 | WMTI | 0.1751 | 0.1749 | 682.7749 | <0.0001 | 0.1266 |
| 4 | Full Multimodal | 0.1279 | 0.1276 | 471.5012 | <0.0001 | 0.0002 |
| 4 | Mean Multimodal | 0.0999 | 0.0997 | 396.8655 | <0.0001 | 0.0672 |
| 4 | BRIA | 0.0880 | 0.0877 | 310.4268 | <0.0001 | -0.0049 |
| 4 | DKI | 0.0688 | 0.0685 | 237.5608 | <0.0001 | 0.0302 |
| 4 | DTI | 0.0696 | 0.0693 | 240.5501 | <0.0001 | -0.1682 |
| 4 | SMT | 0.1040 | 0.1038 | 403.8253 | <0.0001 | -0.0603 |
| 4 | SMT mc | 0.1639 | 0.1637 | 630.6907 | <0.0001 | 0.0803 |
| 4 | WMTI | 0.2239 | 0.2236 | 927.6834 | <0.0001 | 0.1036 |

The table shows predictions of the first four components retrieved from the respective models (as done for brain age predictions, see **Table 2**), using BAG, age, sex, site, as well as age-sex and sex-site interactions as predictors (Equation 1). Both these four components as well as multimodal and approach-specific BAGs are based on data limited to the particular uni- or multi-modal approach and vary therefore in their number of metrics (**Table 2**).

**ST15. Top five diffusion metrics ranked by gain in age prediction accuracy**

| **BRIA** | **DKI** | **DTI** | **SMT** | **mcSMT** | **WMTI** | **Multimodal** |
| --- | --- | --- | --- | --- | --- | --- |
| Micro FA fornix (54957) | MK fornix (39662) | MD fornix  (50535) | MD fornix  (43563) | Intra fornix (38043) | AWF fornix (52531) | Micro FA Fornix (67749) |
| Micro RD right external capsule (22860) | RK fornix (26954) | RD FMIN (18386) | MD right anterior corona radiata (24675) | Extra trans Fornix (35799) | RadEAD ATRL (12328) | RD Fornix right Stria terminalis (17664) |
| Micro FA FMIN  (10081) | AK right anterior limb of internal capsule (16340) | RD fornix right stria terminalis (15431) | MD SLFR (19451) | Extratrans right external capsule (15369) | RadEAD right anterior corona radiata | AK anterior right limb of internalcapsule (17664) |
| Micro FA fornix right stria terminlis  (9853) | AK fornix (10516) | AD fornix (9637) | MD FMIN (13527) | Extra MD anterior left limb of internal capsule (6254) | RadEAD IFOFR (9828) | RadEAD right anterior corona radiata (17375) |
| Micro RD Fornix right stria terminalis (9812) | AK left superior fronto occipital fasciculus (6850) | FA fornix left stria terminalis (9283) | FA fornix (12011) | Extra trans anterior right limb of internal capsule (6126) | RadEAD right external capsule (9793) | RadEAD SLFR (15840) |

Table values can be read as feature name (gain value). Gain refers to the improvement in accuracy brought by a feature to the branches it is on43⁠. Multimodal refers to an approach using the diffusion metrics from all diffusion approaches. Cells containing Fornix are marked in green.

Tracts are abbreviated as follows: ATRL = anterior thalamic radiation left, FMIN = Forceps minor, IFOFR = inferior fronto-occipital fasciculus right, SLFR = superior longitudinal fasciculus right

**ST16. Brain age prediction model performance e*xcluding fornix features* and uncorrected brain age – chronological age correlations comparison**

| **Name** | **MRI features** | **R2** | **RMSE** | **MAE** | **Prediction-Age Correlation** | **Uncorrected Brain Age Correlation Difference to All Data** |
| --- | --- | --- | --- | --- | --- | --- |
| BRIA | 670 | 0.527  (0.010) | 5.131  (0.042) | 4.129  (0.033) | 0.727  [0.722, 0.732] | -0.007*  [-0.009, -0.004] |
| DKI | 201 | 0.550  (0.015) | 5.108  (0.070) | 4.105  (0.065) | 0.742  [0.737, 0.747] | -0.006*  [-0.008, -0.003] |
| DTI | 269 | 0.555  (0.013) | 5.078  (0.066) | 4.079  (0.061) | 0.745  [0.745, 0.750] | -0.005*  [-0.007, -0.003] |
| SMT | 269 | 0.507  (0.008) | 5.347  (0.042) | 4.309  (0.028) | 0.713  [0.707, 0.718] | -0.009*  [-0.011, -0.006] |
| mcSMT | 269 | 0.488  (0.011) | 5.342  (0.045) | 4.303  (0.036) | 0.699  [0.693, 0.705] | -0.015*  [-0.018, -0.012] |
| WMTI | 201 | 0.566  (0.012) | 5.018  (0.062) | 4.031  (0.052) | 0.753  [0.748, 0.757] | -0.003*  [-0.006, -0.001] |
| Mean scores multimodal | 28 | 0.393  (0.012) | 5.932  (0.051) | 4.812  (0.046) | 0.627  [0.621, 0.634] | 0  [-0.0001, 0.0001] |
| Full model multimodal | 1876 | 0.636  (0.012) | 4.591  (0.077) | 3.677  (0.039) | 0.798  [0.794, 0.802] | -0.006*  [-0.007, -0.004] |

In the above, only fornix features are excluded, while QC and all other steps are kept as described in the Methods section. *Importantly*, radiations from the fornix to other tracts such as fornix to stria terminalis radiations were not excluded. Compare results from the full model in Table 2 for uncorrected prediction-age correlations which were the basis for the final column.

Multimodoal mean scores remain unaffected as averaged across the brain.

* p<.001

**ST17. Forceps age-sensitivity**

| **Name** | **Full model Log Likelihood** | **Reduced model Log Likelihood** | **χ2** | **p** | **p_Holm_** |
| --- | --- | --- | --- | --- | --- |
| vintra | 65324.28 | 62344.73 | 5959.09 | <.001 | <.001 |
| vextra | 67328.43 | 66054.27 | 2548.32 | <.001 | <.001 |
| vCSF | 97504.73 | 94607.66 | 5794.14 | <.001 | <.001 |
| microRD | 53988.86 | 48970.28 | 10037.16 | <.001 | <.001 |
| microFA | 78226.20 | 73741.72 | 8968.96 | <.001 | <.001 |
| microAx | 51917.79 | 51008.09 | 1819.40 | <.001 | <.001 |
| microADC | 58051.50 | 53835.77 | 8431.45 | <.001 | <.001 |
| DRADextra | 89573.64 | 86872.66 | 5401.95 | <.001 | <.001 |
| DAXintra | 51663.52 | 49351.15 | 4624.73 | <.001 | <.001 |
| DAXextra | 63746.95 | 61384.28 | 4725.33 | <.001 | <.001 |
| RK | 30223.68 | 28365.61 | 3716.16 | <.001 | <.001 |
| AK | 75898.32 | 74813.14 | 2170.36 | <.001 | <.001 |
| MK | 54795.11 | 52705.19 | 4179.85 | <.001 | <.001 |
| FA | 79855.82 | 74142.48 | 11426.68 | <.001 | <.001 |
| MD | 62580.39 | 58465.88 | 8229.01 | <.001 | <.001 |
| RD | 59656.16 | 54507.04 | 10298.24 | <.001 | <.001 |
| AD | 59783.00 | 58635.84 | 2294.32 | <.001 | <.001 |
| smtFA | 108817.56 | 107246.17 | 3142.78 | <.001 | <.001 |
| smtLong | 272546.89 | 270636.86 | 3820.06 | <.001 | <.001 |
| smtMD | 306783.88 | 303555.26 | 6457.24 | <.001 | <.001 |
| smtTrans | 329895.04 | 326745.72 | 6298.65 | <.001 | <.001 |
| smtMCintra | 62183.23 | 59678.07 | 5010.32 | <.001 | <.001 |
| smtMCextraMD | 300611.95 | 297380.55 | 6462.80 | <.001 | <.001 |
| smtMCextratrans | 292351.49 | 288313.49 | 8075.99 | <.001 | <.001 |
| smtMCdiff | 277388.31 | 277360.92 | 54.77 | <.001 | <.001 |
| AWF | 88642.46 | 85547.48 | 6189.95 | <.001 | <.001 |
| axEAD | 50048.46 | 49980.83 | 135.25 | <.001 | <.001 |
| radEAD | 52979.23 | 48429.12 | 9100.23 | <.001 | <.001 |

**ST18: Comparisons of linear and generalized additive models predicting forceps diffusion metrics**

| **Metric** | **LM AIC** | **GAM AIC** | **LM BIC** | **GAM BIC** | **LM R2adj** | **GAM R2adj** |
| --- | --- | --- | --- | --- | --- | --- |
| vintra | -130624.56 | -130624.65 | -130522.74 | -130531.33 | 0.16 | 0.16 |
| vextra | -134632.86 | -134634.69 | -134531.05 | -134541.36 | 0.08 | 0.08 |
| vCSF | -194985.46 | -194983.16 | -194883.65 | -194889.84 | 0.17 | 0.17 |
| microRD | -107953.72 | -107948.75 | -107851.91 | -107855.43 | 0.26 | 0.26 |
| microFA | -156428.39 | -156426.02 | -156326.58 | -156332.69 | 0.23 | 0.23 |
| microAx | -103811.58 | -103813.40 | -103709.77 | -103720.08 | 0.08 | 0.08 |
| microADC | -116078.99 | -116076.52 | -115977.18 | -115983.19 | 0.23 | 0.23 |
| DRADextra | -179123.28 | -179125.07 | -179021.47 | -179031.74 | 0.15 | 0.15 |
| DAXintra | -103303.03 | -103305.01 | -103201.22 | -103211.69 | 0.14 | 0.14 |
| DAXextra | -127469.89 | -127471.55 | -127368.08 | -127378.22 | 0.14 | 0.14 |
| RK | -60423.37 | -60425.09 | -60321.56 | -60331.76 | 0.13 | 0.13 |
| AK | -151772.64 | -151769.08 | -151670.82 | -151675.76 | 0.09 | 0.09 |
| MK | -109566.23 | -109567.55 | -109464.41 | -109474.23 | 0.12 | 0.12 |
| FA | -159687.65 | -159688.21 | -159585.83 | -159594.89 | 0.28 | 0.28 |
| MD | -125136.77 | -125133.28 | -125034.96 | -125039.96 | 0.22 | 0.22 |
| RD | -119288.32 | -119288.44 | -119186.51 | -119195.11 | 0.26 | 0.26 |
| AD | -119542.00 | -119530.17 | -119440.19 | -119436.84 | 0.09 | 0.09 |
| smtFA | -217611.12 | -217610.05 | -217509.31 | -217516.72 | 0.10 | 0.10 |
| smtLong | -545069.78 | -545071.74 | -544967.97 | -544978.42 | 0.13 | 0.12 |
| smtMD | -613543.77 | -613545.40 | -613441.96 | -613452.07 | 0.18 | 0.18 |
| smtTrans | -659766.09 | -659763.06 | -659664.28 | -659669.73 | 0.17 | 0.17 |
| smtMCintra | -124342.46 | -124343.29 | -124240.65 | -124249.96 | 0.13 | 0.13 |
| smtMCextraMD | -601199.89 | -601200.16 | -601098.08 | -601106.83 | 0.19 | 0.19 |
| smtMCextratrans | -584678.97 | -584678.64 | -584577.16 | -584585.31 | 0.21 | 0.21 |
| smtMCd | -554752.62 | -554754.61 | -554650.81 | -554661.29 | 0.03 | 0.03 |
| AWF | -177260.92 | -177262.82 | -177159.11 | -177169.50 | 0.17 | 0.17 |
| axEAD | -100072.91 | -100063.70 | -99971.10 | -99970.37 | 0.03 | 0.03 |
| radEAD | -105934.46 | -105934.45 | -105832.65 | -105841.12 | 0.25 | 0.24 |

LM = linear model, GAM = generalized additive model, AIC = Akaike information criterion, BIC = Bayesian information criterion. The numbers are derived from the six diffusion approaches’ 28 metrics following Equation 1 for linear models and all variables of the equation allowing splines for non-linear models.
